# Iron intake, body iron status, and risk of breast cancer: a systematic review and meta-analysis

**DOI:** 10.1186/s12885-019-5642-0

**Published:** 2019-06-06

**Authors:** Vicky C. Chang, Michelle Cotterchio, Edwin Khoo

**Affiliations:** 1 Dalla Lana School of Public Health, University of Toronto, 155 College Street, 6th Floor, Toronto, ON M5T 3M7 Canada; 2 Prevention and Cancer Control, Cancer Care Ontario, 620 University Avenue, Toronto, ON M5G 2L7 Canada; 3 Analytics and Informatics, Cancer Care Ontario, Toronto, ON Canada

**Keywords:** Breast cancer, Iron intake, Heme iron, Iron status, Ferritin, Systematic review, Meta-analysis, Dose-response

## Abstract

**Background:**

Iron has been shown to promote breast carcinogenesis in animal models through generation of oxidative stress and interaction with estrogen. Heme iron, which is found exclusively in animal-sourced foods, is suggested to have a more detrimental effect. Epidemiological evidence of the association between iron and breast cancer risk remains inconclusive and has not been comprehensively summarized. This systematic review and meta-analysis evaluated associations between both iron intake and body iron status and breast cancer risk.

**Methods:**

Four electronic databases (MEDLINE, EMBASE, CINAHL, and Scopus) were searched up to December 2018 for studies assessing iron intake and/or biomarkers of iron status in relation to breast cancer risk. Using random-effects meta-analyses, pooled relative risks (RRs) and 95% confidence intervals (CIs) were calculated comparing the highest vs. lowest category of each iron measure. Dose-response meta-analyses were also performed to investigate linear and nonlinear associations.

**Results:**

A total of 27 studies were included in the review, of which 23 were eligible for meta-analysis of one or more iron intake/status measures. Comparing the highest vs. lowest category, heme iron intake was significantly associated with increased breast cancer risk, with a pooled RR of 1.12 (95% CI: 1.04–1.22), whereas no associations were found for dietary (1.01, 95% CI: 0.89–1.15), supplemental (1.02, 95% CI: 0.91–1.13), or total (0.97, 95% CI: 0.82–1.14) iron intake. Associations of iron status indicators with breast cancer risk were generally in the positive direction; however, a significant pooled RR was found only for serum/plasma levels (highest vs. lowest) of iron (1.22, 95% CI: 1.01–1.47), but not for ferritin (1.13, 95% CI: 0.78–1.62), transferrin saturation (1.16, 95% CI: 0.91–1.47), or total iron-binding capacity (1.10, 95% CI: 0.97–1.25). In addition, a nonlinear dose-response was observed for heme iron intake and serum iron (both *P*_nonlinearity_ < 0.05).

**Conclusions:**

Heme iron intake and serum iron levels may be positively associated with breast cancer risk. Although associations were modest, these findings may have public health implications given the widespread consumption of (heme) iron-rich foods. In light of methodological and research gaps identified, further research is warranted to better elucidate the relationship between iron and breast cancer risk.

**Electronic supplementary material:**

The online version of this article (10.1186/s12885-019-5642-0) contains supplementary material, which is available to authorized users.

## Background

Iron is an essential nutrient required for many biological processes in the human body, such as oxygen transport, DNA synthesis, and energy production [1]. However, owing to its strong capacity to both accept and donate electrons, iron also readily participates in reduction-oxidation (redox) reactions that lead to the generation of reactive oxygen species (ROS) and subsequent oxidative damage to tissues and cellular components, particularly DNA, proteins, and lipids [2, 3]. As such, both high dietary iron intake and elevated body iron status have been hypothesized to increase the risks of several cancers, including breast cancer [4–9]. Notably, the World Health Organization’s International Agency for Research on Cancer has identified “iron (in food and as supplements)” as one of the “high priority” agents or exposures to be assessed in relation to cancer risk [10].

In the general, non-transfused population, iron is obtained almost exclusively from the diet, either in the form of heme or non-heme iron [11, 12]. Heme iron is the organic form of iron derived only from animal source foods, including meat, poultry, and fish/seafood [12]. On average, heme iron constitutes approximately 40% of the total iron content in cooked meats, with the highest levels found in red meat (e.g., beef, pork), although concentrations may further vary according to differences in meat type/cut, cooking or preparation method, and meat doneness level [12–14]. While also present in animal sources, non-heme iron constitutes all of the iron content in plant-based foods, including vegetables, fruits, and legumes, as well as iron-fortified products (e.g., cereals) [12]. Heme iron may be of particular concern with respect to cancer risk due to its greater bioavailability and involvement in the formation of carcinogenic *N*-nitroso compounds [15, 16]. Once absorbed by intestinal cells and exported into circulation, iron is bound by its transport protein transferrin and delivered to tissues and cells, where it is either used or stored by binding to ferritin [11]. Common indicators of body iron status include circulating (serum or plasma) levels of ferritin, iron, transferrin, transferrin receptor (TfR), total iron-binding capacity (TIBC), and transferrin saturation (TSAT) [17, 18]. Iron status may also be assessed using nail [19], hair [20], and tissue [17, 21] samples.

Iron is suggested to have a role in breast cancer development through its interaction with estrogen in oxidative stress and other pathways [22]. For example, iron catalyzes the redox cycling of catechol estrogen metabolites to form quinones and semiquinones, which have been shown to stimulate ROS production and contribute to breast carcinogenesis in cell cultures and in vivo [7, 8, 23]. In addition, superoxide radicals generated from estrogen redox cycling may trigger the release of free iron (i.e., the more biologically active ferrous [Fe^2+^] form) from ferritin storage, further amplifying oxidative stress and inducing DNA damage [7, 24]. Importantly, the role of iron in breast cancer is strongly supported by animal experiments demonstrating that excess iron through diet or subcutaneous injection promotes the initiation and growth of mammary tumours in rodents [25–28].

Despite strong biological plausibility and evidence from animal studies, epidemiological evidence of the association between iron and breast cancer risk in humans is inconsistent, inconclusive, and has not been adequately summarized. Although several narrative reviews have discussed iron’s role in breast cancer, they focused largely on biological mechanisms and only presented selected epidemiologic findings [4–9]. A 2014 systematic review/meta-analysis on iron and cancer risk by Fonseca-Nunes et al. identified a total of 59 studies published between 1995 and 2012, of which only seven studies assessed iron intake, and none assessed body iron status, in relation to breast cancer risk [29]. Overall, the review concluded that heme iron intake may be positively (albeit not significantly for breast cancer) associated with cancer risk, whereas biomarkers of iron status, such as serum ferritin, may be negatively associated with cancer risk [29]. However, the review was limited in terms of its search strategy (e.g., single database searched, missing relevant search terms), lacked information on study quality assessment, and provided a primarily qualitative synthesis of findings, with meta-analysis conducted for heme iron intake only.

Given the growing body of literature on iron intake/status and breast cancer risk, an updated, comprehensive, and quantitative review focusing specifically on this topic is warranted. We conducted a systematic review and meta-analysis of epidemiologic studies to evaluate associations between different types of iron intake, as well as indicators of body iron status, and risk of breast cancer.

## Methods

This systematic review and meta-analysis was conducted and reported with reference to the Preferred Reporting Items for Systematic Reviews and Meta-Analyses (PRISMA) [30] and the Meta-analysis Of Observational Studies in Epidemiology (MOOSE) [31] guidelines and checklists.

### Data sources and search strategy

Systematic electronic database searches were conducted using MEDLINE, EMBASE, CINAHL, and Scopus to identify studies published up to December 31, 2018 that investigated the association between iron intake/status and breast cancer risk, without any language restrictions. The search included a combination of Medical Subject Headings terms, keywords, and variations of text words related to iron (e.g., “iron”, “Fe”, “ferric”, “ferrous”, “ferritin”, “transferrin”, “TfR”, or “TIBC”) and breast cancer (e.g., “breast”, “mammary”, or “nipple”, combined with “cancer”, “neoplasm”, “tumor”, “carcinoma”, “adenocarcinoma”, or “malignancy”). The full electronic search strategy is presented in Additional file 1. To identify additional potentially eligible studies, reference lists of all included studies and relevant review articles were also hand-searched.

### Eligibility criteria and study selection

Studies were eligible for inclusion if they: 1) involved human subjects; 2) were primary research studies; 3) utilized a cohort or case-control design, including traditional case-control, nested case-control, and case-cohort studies; 4) assessed any prediagnostic measure of *iron intake* and/or *body iron status* as an exposure (see below section on “Exposure definitions” for details); 5) examined breast cancer as an outcome in females; and 6) reported (or provided sufficient data to calculate) an odds, risk, or hazard ratio for the association between iron intake/status and breast cancer risk.

Animal and cell culture studies, non-primary studies (e.g., reviews, editorials, letters to editor), conference abstracts without full-text, case reports, case series, cross-sectional studies, ecological studies, and studies combining female and male breast cancer were excluded. We also excluded studies assessing postdiagnostic levels of iron intake (i.e., studies specifically asking about diet or supplement use after diagnosis) or body iron status (i.e., studies where biological samples were collected after diagnosis), since these measures may be influenced by breast cancer pathogenesis and treatment [32, 33] and are thus less relevant for evaluating the role of iron in relation to breast cancer risk.

Following removal of duplicate records, titles and abstracts of citations retrieved from the electronic databases were screened to identify potentially relevant studies. Full-texts of these identified studies were then obtained and assessed in detail for inclusion or exclusion. Both title/abstract screening and full-text eligibility assessment were performed independently by two authors (VCC and EK) using the web-based systematic review tool Covidence (Veritas Health Innovation, Melbourne, Australia) [34]. Any disagreement was resolved through discussion and consensus, and all authors approved the final list of studies included.

### Exposure definitions

In this review, measures of *iron intake* were classified and defined as below: dietary iron (iron from foods alone), supplemental iron (iron from single-ingredient iron supplements and/or iron-containing multivitamin/mineral supplements), total iron (sum of dietary and supplemental iron), heme iron (iron estimated from animal-based foods as described in the original studies, e.g., 40% of total iron from meat, literature-based meat-specific percentages [13], laboratory-based heme iron database [14]), and non-heme iron (total dietary iron minus heme iron).

The following serum or plasma indicators of *body iron status* were included when available: ferritin (marker of body iron stores), iron (circulating iron bound to transferrin), transferrin (direct measure of circulating transferrin available to bind iron), TIBC (total amount of iron that can be bound by circulating transferrin, i.e., indirect or proxy measure of transferrin), TSAT (percentage of iron-binding sites on transferrin that are occupied by iron, typically calculated as the ratio of serum iron to TIBC or serum iron to transferrin), and TfR (indicator of balance between cellular iron demand and supply) [17, 18]. In addition, finger/toenail and hair iron, which may reflect longer-term exposure [19, 20], as well as tissue (e.g., bone marrow, liver, breast) iron [17, 21] were also considered. Higher levels of each biomarker are associated with higher iron status, with the exceptions of transferrin, TIBC, and TfR, which are inversely related to iron status [17, 18].

### Data extraction

The following information was extracted from each included study: author name, publication year, country of study conduct, study name, study design, study period and setting, duration of follow-up (where applicable), sample size (number of cases/total number of participants for cohort studies; number of cases/controls for case-control studies), population characteristics (age and menopausal status), measure(s) of iron intake/status reported and their methods of assessment, breast cancer case ascertainment, effect estimates and corresponding 95% confidence intervals (CIs), variables matched or adjusted for in the analysis, and any information needed for study quality assessment. Where available, results stratified by menopausal status (premenopausal and postmenopausal) at breast cancer diagnosis and hormone receptor (estrogen receptor [ER]/progesterone receptor [PR]) tumour subtype were also extracted.

Data extraction was performed by one author (VCC) and verified independently by another (EK). For studies with missing information, we referred to related publications (e.g., detailed reports of study design and population characteristics) or contacted the corresponding author of the original study for clarification or additional information.

### Quality assessment

The quality of included studies was assessed independently by two authors (VCC and EK) using the Newcastle-Ottawa Scale (NOS) [35], with any disagreement resolved by discussion and consensus. The NOS includes study design-specific items for cohort and case-control studies and evaluates three broad domains of bias: 1) selection of study subjects; 2) comparability of groups (i.e., control for potential confounding factors); and 3) ascertainment of the exposure or the outcome [35]. If a study examined the association of both iron intake and iron status with breast cancer risk, its quality was assessed separately for each type of exposure because of possible differences in confounding control and/or biases related to exposure ascertainment. The NOS yields a score ranging from 0 (lowest) to 9 (highest) [35]. In this review, studies with scores of 7 or greater were considered high-quality, while those scoring below 7 were considered low-quality. Detailed NOS coding manuals are presented in Additional file 2.

### Statistical analysis

Meta-analyses of the associations between iron intake/status and breast cancer risk were performed separately for each subtype of iron intake or iron status indicator with at least two available studies. When multiple publications reported data on the same iron measure from identical or overlapping study populations, only the publication with the largest sample size or longest duration of follow-up was included in the meta-analysis for the specific iron measure.

For each subtype of iron intake or iron status indicator, the pooled relative risk (RR) was used as the summary measure of association and was estimated by combining odds, risk, and hazard ratios reported by individual studies. Odds, risk, and hazard ratios, hereafter all referred to as RRs, were assumed to be equivalent in our analyses given that breast cancer is a relatively rare disease outcome (i.e., less than 10%) [36]. If a study reported RRs and 95% CIs from two or more regression models with different levels of covariate adjustment, estimates from the most fully adjusted model were used in the analyses. To account for within- and between-study variability, pooled RRs and corresponding 95% CIs were computed using the DerSimonian and Laird (DL) random-effects model [37]. Additionally, pooled RRs and 95% CIs were also calculated using the profile likelihood random-effects model as the DL method has been suggested to overestimate precision when there is a small number of studies [38]; however, since the two models yielded very similar results and led to the same conclusions for all iron measures, estimates from the DL model (most common method) were presented.

In our main analysis, the pooled RR comparing the highest to the lowest category of each iron intake/status measure was computed. For supplemental iron intake, we examined the dichotomous measure “use vs. no use” instead, as only one study reported RRs across doses of supplemental iron [39]; for that study, the adjusted RR for “use” (all categories > 0 mg/day) vs. “no use” (0 mg/day) was estimated using the method described by Hamling et al., which involves the reconstruction of contingency tables to calculate the adjusted effect estimates and their CIs [40]. For one iron biomarker study where the reference category was not the lowest [41], the adjusted RR comparing the highest vs. lowest category was calculated also using the Hamling method [40]. For one study that reported RR for each 1-standard deviation (SD) increase in iron intake [42], we converted the RR such that it corresponded to a comparison for the highest vs. lowest quartile; this was done by multiplying the natural logarithm of the original RR by 2.54 and exponentiating the product, under the assumption of a standard normal distribution where the difference in means between the highest and lowest quartiles is 2.54 SDs [43].

To investigate linear and nonlinear dose-response relations between iron intake/status and breast cancer risk, we further conducted random-effects dose-response meta-analyses using a generalized least-squares method for trend estimation, as proposed by Greenland and Longnecker [44] and Orsini et al. [45, 46]. To prepare the data for these analyses, RRs and 95% CIs across at least three categories of the exposure (iron intake or status) were obtained from each study, along with exposure values (i.e., dose) and numbers of cases/non-cases for each category [44, 45]. Whenever reported, the mean or median value of iron intake or iron biomarker level for each category was assigned as the “dose” corresponding to each RR estimate; otherwise, the midpoint (calculated as the average of the maximum and minimum values for each category) was used. If a study did not report the maximum or minimum value for the highest or lowest category, respectively, the midpoint was calculated by assuming the range of that category to be the same as that of the adjacent category. When units of measurement for a specific exposure differed across studies, they were converted to the most commonly reported or conventional unit. For example, when iron intake was reported in mg/1000 kcal, we converted it to mg/day using the mean total energy intake (kcal/day) provided by the study. Similarly, serum iron concentration reported in μmol/L was converted to μg/dL by multiplying by 5.5866 (1 μg/dL = 0.179 μmol/L iron) [47]. If the number of cases/non-cases across exposure categories was not available, it was estimated by dividing the total number of subjects (or person-years; for cohort studies) or controls (for case-control studies) by the total number of categories (assuming nearly equal distribution across quantiles); the number of cases was then estimated accordingly based on the RRs. In addition to meta-analysis assuming a linear trend (e.g., pooled RR per unit increase in iron intake) [45], we examined potential nonlinear associations using restricted cubic splines analyses with three knots (located at the 10th, 50th, and 90th percentiles), and the presence of nonlinearity was assessed by testing the significance of the coefficient for the second spline [46].

Heterogeneity between studies was assessed using the Cochran’s Q test (*P* < 0.10 considered statistically significant) and the I^2^ statistic quantifying the proportion of the total variability attributable to heterogeneity [48]; I^2^ values of 25, 50, and 75% roughly indicate low, moderate, and high heterogeneity, respectively [49]. To explore potential effect modification and sources of heterogeneity, subgroup analyses were performed according to study design (cohort or case-control), geographic location (North America, Europe, Asia, or Australia), menopausal status (premenopausal or postmenopausal), study quality (NOS score ≥ 7 or < 7), dietary assessment method (structured interview or self-administered questionnaire), biological sample (serum or plasma), and adjustments for specific confounders, including body mass index (BMI), physical activity, alcohol intake, oral contraceptive (OC) and/or hormone replacement therapy (HRT) use, and family history of breast cancer. Where at least 10 studies were available, univariable meta-regression was performed on each of the aforementioned variables to further assess their influence on heterogeneity, with *P* < 0.10 indicating statistical significance [48]. Notably, although pre-specified, subgroup analyses were not conducted by breast cancer tumour (ER/PR) status, as there were less than two studies reporting these results for each iron measure.

Publication bias was evaluated using funnel plots and Begg’s rank-correlation [50] and Egger’s regression [51] tests (*P* < 0.10 considered statistically significant). Finally, influence of individual studies was investigated by recalculating the pooled RR and 95% CI each time a single study was omitted from the analysis.

Analyses were performed using Stata/MP, version 14 (StataCorp LP, College Station, TX, USA). Statistical tests were two-sided, with statistical significance evaluated at *P* < 0.05 unless otherwise specified.

## Results

### Search results

**Fig. 1:**
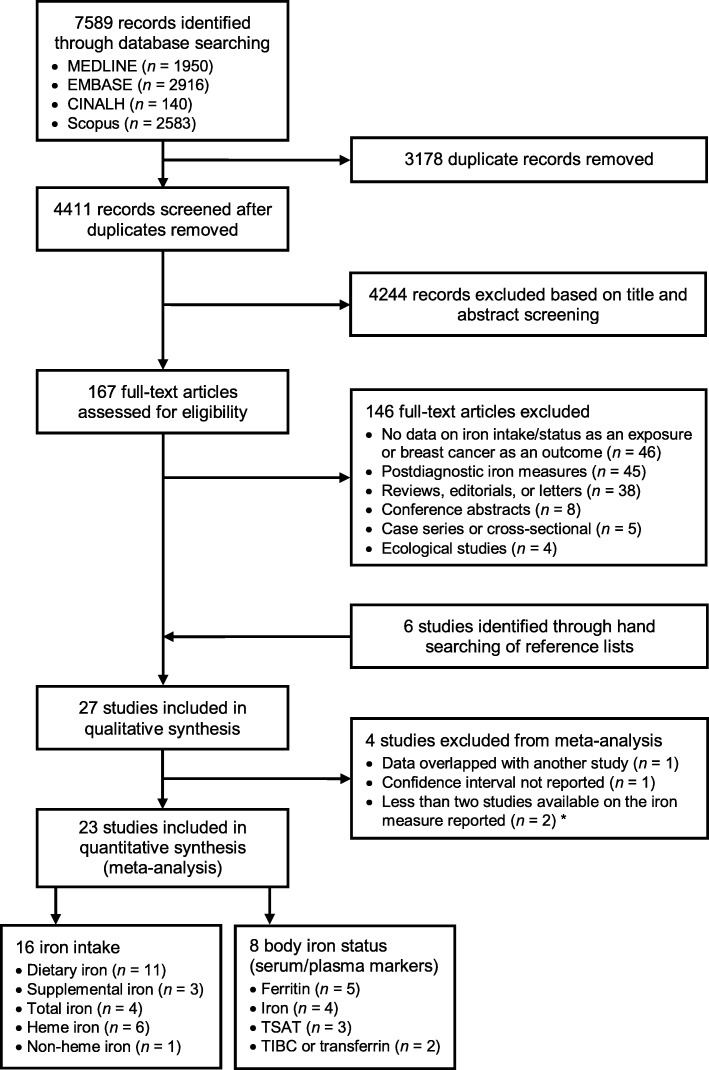
Flow diagram of study selection for the systematic review and meta-analysis. *One study reporting on toenail iron [69] and the other on breast tissue iron [70] as the only iron measure

Our search initially yielded 7589 records. After duplicates were removed, titles and abstracts of 4411 articles were screened, of which 167 full-texts were further assessed for eligibility. Twenty-seven studies, including 17 studies examining iron intake [39, 42, 52–66] and 11 studies examining body iron status [41, 61, 67–75] in relation to breast cancer risk, met the inclusion criteria of our systematic review. Several studies reported data on multiple measures of iron intake and/or status and were included in more than one meta-analysis. Four of the 27 studies were excluded from all meta-analyses but remained in the review, including one assessing adolescent intakes of total and heme iron [64] in the same (but a smaller subset of) study cohort as another study assessing adult intakes of total and heme iron [63], one iron status study where CIs for the RRs were not reported [67], and two studies that were the only ones analyzing toenail [69] or breast tissue [70] iron. A flow diagram detailing the study selection process is presented in Fig. 1.

### Study characteristics and quality

Table 1 summarizes the main characteristics and findings of included studies. Among all 27 studies reviewed, the year of publication ranged from 1990 to 2018, with six studies published before 2000 [52–54, 67–69], nine studies between 2000 and 2009 [39, 55–61, 70], and 12 studies in 2010 or later [41, 42, 62–66, 71–75]. The majority of studies were conducted in the United States (*n* = 12) [39, 42, 57, 58, 62–64, 66, 68–70, 73] or Canada (*n* = 1) [59], while the rest were in Europe (*n* = 9; including two in Germany and one in each of Denmark, Italy, the United Kingdom, Switzerland, France, Finland, and Sweden) [52–56, 65, 67, 72, 75], Asia (*n* = 4; including two in China, one in Taiwan, and one in Japan) [41, 60, 61, 71], and Australia (*n* = 1) [74].

**Table 1:** Summary of studies investigating associations between iron intake and iron status and breast cancer risk

Author and year [ref], country	Study design and study period/setting; duration of follow-up	Population (no. of cases/total or cases/controls)^a^	Age range and percent postmenopausal	Iron intake or status assessment	Iron intake or status measure (unit) and comparison	Main results^b^ RR (95% CI)	Included in meta-analysis	Adjusted or matched variables	NOS score
Iron intake (*n* = 17)									
Ewertz and Gill 1990 [52], Denmark	Population-based case-control (1983–1984)	All women (1486/1336)	Cases: < 70 y, 56%; Controls: < 70 y, 59%	21-item, self-administered FFQ + supplement use questions (pilot-tested against interviews) assessing usual intake in the year prior to diagnosis	Iron supplement use: yes vs. no	All: 1.14 (0.65–2.02)	Yes	Age, place of residence	7
Negri et al. 1996 [53], Italy	Hospital-based case-control (multicentre study in six Italian areas, 1991–1994)	All women (2569/2588)	Cases: 23–74 y, 61%; Controls: 20–74 y, 67%	78-item, validated, interviewer-administered FFQ assessing usual intake in the 2 years before diagnosis	Dietary iron (mg/day, energy-adjusted using the residual method): highest (> 16.52) vs. lowest (≤10.49) quintile	All: 0.85 (0.7–1.0)	Yes	Age, study centre, education, parity, energy intake, alcohol intake	6
Cade et al. 1998 [54], UK	Hospital-based case-control (UK breast screening programme clinics in southern England, 1990–1992)	All women (220/825)	Cases: 50–65 y, 86%; Controls: 50–65 y, 80%	25-item, interviewer-administered FFQ (completed before mammogram results were known) and 141-item, validated, self-administered FFQ (completed after clinic visit) assessing usual intake over the past year	Dietary iron (mg/day, crude): highest (NR) vs. lowest (NR) quartile	All: 0.49 (0.23–1.01)	Yes	Age, age at menarche, age at first birth, social class, BMI, smoking, components of calorie intake (alcohol, complex carbohydrates, protein, polyunsaturated fat, monounsaturated fat, saturated fat, cholesterol, sugar), non-caloric nutrients (vitamin E)	5
Levi et al. 2001 [55], Switzerland	Hospital-based case-control (single-centre, 1993–1999)	All women (289/442)	Cases: < 75 y, 69%; Controls: 23–74 y, 65%	79-item, validated, interviewer-administered FFQ assessing usual intake in the 2 years before diagnosis	Dietary iron (mg/day, crude): highest (median 16.8) vs. lowest (median 9.0) tertile	All: 1.21 (0.65–2.26)	Yes	Age, education, parity, menopausal status, BMI, total energy intake, alcohol drinking	6
Adzersen et al. 2003 [56], Germany	Hospital-based case-control (single-centre, 1998–2000)	All women (310/353)	Cases: 25–75 y, 55%; Controls: 25–75 y, 57%	161-item, validated, self-administered FFQ assessing usual intake in the year before hospital admission	Dietary iron (mg/day, crude): highest (> 14.3) vs. lowest (< 9.0) quartile	All: 0.66 (0.32–1.33)	Yes	Age, total energy intake without alcohol, age at menarche, age at first birth, age at menopause, mother/sister with breast cancer, current smoking, history of BBD and/or operation, BMI, alcohol intake, current HRT or HRT during the past year	5
Michels et al. 2006 [57], USA	Nested case-control (Nurses’ Mothers’ Study, nested in Nurse’s Health Study I and II, 1976–1993)	All women (582/1569)	25–55 y at cohort enrolment; 27% postmenopausal at diagnosis (cases)	30-item, validated, self-administered FFQ assessing diet during preschool age (3–5 y); completed by participants’ mothers after case diagnosis	Dietary iron (mg/day, energy-adjusted using the residual method): highest (mean 7.23) vs. lowest (mean 2.54) quintile	All: 0.79 (0.55–1.13)	Yes	Age, age at menarche, parity, age at first birth, family history of breast cancer, adult BMI, total energy intake	6
Hong et al. 2007 [58], USA	Nested case-control (American Cancer Society Cancer Prevention Study II Nutrition Cohort, 1992–2001); 10 y	Postmenopausal (502/505)	50–74 y at cohort enrolment; 100% postmenopausal at diagnosis	68-item, validated, self-administered FFQ assessing usual intake over the past year; completed at baseline of the original cohort study	Total iron (mg/day, energy-adjusted using the residual method): highest (> 22.5) vs. lowest (≤9.6) tertile	Post: 1.06 (0.77–1.47)^c^	Yes	Age, family history of breast cancer, HRT, BMI, age at menarche, age at menopause, smoking status, race, parity (crude RRs calculated from raw tabulated data)	7
					Iron-containing multivitamin supplement use: yes vs. no	Post: 1.13 (0.87–1.49)^c^	Yes		
Kabat et al. 2007 [59], Canada	Prospective cohort (Canadian National Breast Screening Study, 1982–2000); mean 16.4 y	All women (2491/48662) Premenopausal cases: 1171 Postmenopausal cases: 993	40–59 y and 37% postmenopausal at baseline	86-item, validated, self-administered FFQ assessing usual intake reported at baseline	Dietary iron (mg/day, energy-adjusted using the residual method): highest (≥14.99) vs. lowest (< 11.90) quintile	All: 0.97 (0.85–1.10) Pre: 1.07 (0.89–1.30) Post: 0.87 (0.71–1.06)	Yes	Age, BMI, menopausal status, parity, age at menarche, family history of breast cancer in a first-degree relative, history of BBD, OC use, HRT, total energy intake, alcohol intake, education, study centre, randomisation group in the original trial	8
					Heme iron (mg/day, energy-adjusted using the residual method): highest (> 2.95) vs. lowest (< 1.58) quintile	All: 1.03 (0.90–1.18) Pre: 1.03 (0.84–1.25) Post: 0.97 (0.78–1.20)	Yes		
Kallianpur et al. 2008 [60], China	Population-based case-control (Shanghai Breast Cancer Study I and II, 1996–1998 and 2002–2005)	All women (3452/3474) Premenopausal (2086/1968) Postmenopausal (1366/1506)	Cases: 25–70 y, 40%; Controls: 25–70 y, 43%	76-item, validated, interviewer-administered FFQ assessing usual intake over the past 5 years, ignoring any recent changes	Dietary iron (mg/day, crude): highest (NR) vs. lowest (NR) quartile	All: 1.31 (0.96–1.78) Pre: 1.30 (0.86–1.97) Post: 1.33 (0.83–2.14)	Yes	Age, education, BMI, waist-to-hip ratio, age at menarche, age at first live birth, family history of breast cancer, regular exercise, total energy intake, study phase, age at menopause, vitamin A, vitamin C, vitamin E, folic acid, isoflavone intake, vitamin supplement use, saturated fat and monounsaturated fat intake	8
					Animal-derived (largely heme) iron (mg/day, crude): highest (NR) vs. lowest (NR) quartile	All: 1.50 (1.19–1.88) Pre: 1.61 (1.20–2.15) Post: 1.42 (0.98–2.04)	Yes (as a proxy for heme iron)		
					Plant-derived (non-heme) iron (mg/day, crude): highest (NR) vs. lowest (NR) quartile	All: 0.99 (0.75–1.29) Pre: 0.96 (0.67–1.36) Post: 1.02 (0.67–1.56)	No (only study that reported this measure)		
Ferrucci et al. 2009 [39], USA	Prospective cohort (Prostate, Lung, Colorectal, and Ovarian [PLCO] Cancer Screening Trial, 1998–2006); mean 5.5 y	Postmenopausal (1205/52158)	55–74 y and 100% postmenopausal at baseline	124-item, validated, self-administered FFQ assessing usual intake over the past year; completed at baseline	Total iron (mg/day, energy-adjusted using the residual method): highest (> 31.2) vs. lowest (≤11.4) quintile	Post: 1.08 (0.90–1.30)	Yes	Age, race, education, study centre, randomisation group, family history of breast cancer, age at menarche, age at menopause, age at first birth and number of live births, history of BBD, number of mammograms during past 3 years, menopausal HRT use, BMI, alcohol intake, total fat intake, total energy intake	7
					Dietary iron (mg/1000 kcal, energy-adjusted by nutrient density): highest (> 10.3) vs. lowest (≤6.9) quintile	Post: 1.25 (1.02–1.52)	Yes		
					Heme iron (mg/1000 kcal, energy-adjusted by nutrient density): highest (> 0.23) vs. lowest (≤0.07) quintile	Post: 1.12 (0.92–1.38)	Yes		
					Supplemental iron (mg/day): highest (21.4–39.4) vs. lowest (0) category	Post: 1.00 (0.74–1.35)	Yes		
Moore et al. 2009 [61], China	Nested case-control (Breast Self Examination Trial cohort, 1989–2000)	All women (248/1040)	30–63 y at cohort enrolment; 35% postmenopausal at diagnosis	115-item, validated, interviewer-administered FFQ assessing usual intake in adult life; completed prior to biopsy	Dietary iron (mg/day, crude): highest (> 17.5) vs. lowest (≤12.0) quartile	All: 0.96 (0.53–1.77)	Yes	Age, year of interview, total energy intake, dietary vitamin C intake	4
Kabat et al. 2010 [62], USA	Prospective cohort (National Institutes of Health [NIH]–AARP Diet and Health Study, 1995–2003); 6.5 y	Postmenopausal (3396/116674)	50–71 y and 100% postmenopausal at baseline	124-item, validated, self-administered FFQ assessing usual intake over the past year (completed at baseline) and a second FFQ with meat-cooking module (within 6 months after initial FFQ)	Dietary iron (mg/1000 kcal, energy-adjusted by nutrient density): highest (≥10.1) vs. lowest (< 6.8) quintile	Post: 1.02 (0.90–1.15)	Yes	Age, BMI, age at menarche, age at first live birth, family history of breast cancer, menopausal HRT, education, race, total energy intake, total fat intake, total fibre intake, alcohol intake, physical activity, smoking, age at menopause, number of breast biopsies	7
					Heme iron (μg/1000 kcal, energy-adjusted by nutrient density): highest (≥216.7) vs. lowest (< 62.9) quintile	Post: 1.01 (0.89–1.14)	No (analysis based on a subset of a larger cohort [66])		
Bradshaw et al. 2013 [42], USA	Population-based case-control (Long Island Breast Cancer Study Project, 1996–1997)	All women (1463/1500)	20–98 y, 67% postmenopausal	101-item, validated, self-administered FFQ assessing usual intake 1 year prior to study interview	Total iron (mg/day, crude): per 1-SD (4.41) increase; highest (> 12.3) vs. lowest (< 7.1) quartile	All: 1.10 (0.71–1.72); 1.27 (0.42–3.96)^c^	Yes	Age, total energy intake, carbohydrates, calcium, fibre, magnesium, zinc, alpha-carotene, beta-carotene, cryptoxanthin, lutein, lycopene, oleic acid, pro-alpha carotenes, vitamin C, vitamin E, riboflavin, cobalamin, pyridoxine, folate, betaine, free choline, glycerophosphocholine, methionine, free phosphocholine, phosphotidylcholine, sphingomyelin, anthocyanidins, flavan-3-ols, flavanones, flavones, flavonols, isoflavones, lignans	6
Farvid et al. 2014 [63], USA	Prospective cohort (Nurses’ Health Study II, 1991–2011); 20 y	All women (2830/88803) Premenopausal cases: 1511 Postmenopausal cases: 918	26–45 y and 0% postmenopausal (i.e., 100% premenopausal) at baseline	130-item, validated, self-administered FFQ assessing usual intake over the past year; completed at baseline	Total iron (mg/day, energy-adjusted using the residual method): All: highest (median 50.9) vs. lowest (median 10.2) quintile Pre: highest (median 50.9) vs. lowest (median 10.2) quintile Post: highest (median 44.4) vs. lowest (median 10.2) quintile	All: 0.85 (0.75–0.96) Pre: 0.88 (0.74–1.04) Post: 0.83 (0.68–1.01)	Yes	Age, race, family history of breast cancer in mother or sisters, history of BBD, smoking, height, BMI, age at menarche, parity and age at first birth, OC use, alcohol intake, energy intake, HRT use, menopausal status, age at menopause	7
					Heme iron (mg/day, energy-adjusted using the residual method): All: highest (median 1.6) vs. lowest (median 0.6) quintile Pre: highest (median 1.6) vs. lowest (median 0.6) quintile Post: highest (median 1.7) vs. lowest (median 0.7) quintile	All: 1.12 (0.99–1.28) Pre: 1.15 (0.97–1.37) Post: 0.96 (0.79–1.17)	Yes		
Farvid et al. 2015 [64], USA	Prospective cohort (Nurses’ Health Study II, 1998–2011); 13 y	All women (1132/44231) Premenopausal cases: 546 Postmenopausal cases: 483	33–52 y at baseline	124-item, validated, self-administered FFQ assessing diet during adolescence (i.e., 1960–1980); completed in 1998 (start of follow-up)	Total iron (mg/day, energy-adjusted using the residual method): All: highest (median 17.5) vs. lowest (median 11.7) quintile Pre: highest (median 17.6) vs. lowest (median 11.8) quintile Post: highest (median 16.7) vs. lowest (median 11.6) quintile	All: 0.88 (0.72–1.07) Pre: 0.88 (0.65–1.18) Post: 0.72 (0.54–0.97)	No (analysis based on a subset of a larger cohort [63])	Age, race, family history of breast cancer in mother or sisters, history of BBD, smoking, height, weight gain since age 18, BMI at age 18, age at menarche, parity and age at first birth, OC use, adolescent alcohol intake, adult alcohol intake, adolescent energy intake, HRT use, menopausal status, age at menopause	7
					Heme iron (mg/day, energy-adjusted using the residual method): All: highest (median 2.6) vs. lowest (median 1.0) quintile Pre: highest (median 2.5) vs. lowest (median 1.0) quintile Post: highest (median 2.6) vs. lowest (median 1.1) quintile	All: 1.01 (0.83–1.22) Pre: 1.14 (0.86–1.51) Post: 0.92 (0.69–1.22)	No (analysis based on a subset of a larger cohort [63])		
Diallo et al. 2016 [65], France	Prospective cohort (Supplémentation en Vitamines et Minéraux Antioxydants [SU.VI.MAX] trial, 1994–2007); median 12.6 y	All women (188/4646) Premenopausal cases: 59 Postmenopausal cases: 129	35–60 y and 30% postmenopausal at baseline	Repeated 24-h dietary records administered via a telephone-based terminal; completed every 2 months during the first 2 years of follow-up (intake averaged from ≥3 valid records)	Dietary iron (mg/day, crude): highest (> 11.9) vs. lowest (< 9.3) tertile	All: 1.67 (1.02–2.71) Pre: 1.39 (0.58–3.29) Post: 1.85 (1.02–3.34)	Yes	Age, energy intake without alcohol, intervention group of the initial SU.VI.MAX trial, number of 24-h dietary records, smoking status, education, physical activity, height, BMI, alcohol intake, family history of breast cancer, lipid intake, HRT use, number of children; additionally adjusted for OC use, heavy period, and use of hormonal intrauterine system in premenopausal women	8
					Iron from red meat (mg/day, crude): highest (NR) vs. lowest (NR) tertile	All: 1.00 (0.70–1.43)	Yes (as a proxy for heme iron)		
Inoue-Choi et al. 2016 [66], USA	Prospective cohort (NIH-AARP Diet and Health Study, 1995–2006); mean 9.4 y	Postmenopausal (9305/193742)	50–71 y and 100% postmenopausal at baseline	124-item, validated, self-administered FFQ assessing usual intake over the past year; completed at baseline	Heme iron (μg/1000 kcal, energy-adjusted by nutrient density): highest (median 303.5) vs. lowest (median 44.2) quintile	Post: 1.11 (1.03–1.19) ER+/PR+: 1.07 (0.94–1.21) ER−/PR–: 1.06 (0.83–1.37)	Yes	Age, race, BMI, height, education, smoking, alcohol intake, physical activity, family history of breast cancer, age at menarche, age at menopause, age at first live birth, number of live births, HRT use, OC use, number of previous breast biopsy, total energy intake, total fat intake, fibre intake	7
Body iron status (*n* = 11)									
Knekt et al. 1994 [67], Finland	Prospective cohort (Finnish Mobile Clinic Health Examination Survey, 1966–1984); mean 14 y	All women (192/18813)	20–74 y at baseline	Serum; Technicon AutoAnalyzer (colorimetric method)	Serum iron (μg/dL): highest (> 125) vs. lowest (≤69) quartile	All: 0.85 (NR), trend not significant	No (CI missing)	Age, smoking	7
					Serum TIBC (μg/dL): highest (> 388) vs. lowest (≤313) quartile	All: 1.00 (NR), trend not significant	No (CI missing)		
					Serum TSAT (%): highest (> 36.3) vs. lowest (< 20.0) quartile	All: 0.78 (NR), trend not significant	No (CI missing)		
Herrinton et al. 1995 [68], USA	Prospective cohort (Kaiser Permanente Northern California Multiphasic Health Checkup cohort, 1969–1990); mean 17.6 y	All women (900/28150)	20–84 y (39% aged ≥50 y) at baseline	Serum; Autochemist multichannel analyzer (colorimetric method)	Serum TSAT (%): highest (≥34.5) vs. lowest (≤20.3) quartile	All: 1.10 (0.88–1.30)	Yes	Age, race	7
Garland et al. 1996 [69], USA	Nested case-control (Nurses’ Health Study, 1982–1987); 4 y	All women (433/459) Premenopausal (193/190) Postmenopausal (208/241)	36–61 y at baseline (toenail collection)	Toenail; instrumental neutron activation analysis	Toenail iron (μg/g): highest (> 50.8) vs. lowest (< 20.3) quintile	All: 0.89 (0.56–1.40) Pre: 0.45 (0.21–0.95) Post: 1.56 (0.80–3.03)	No (only study that reported this measure)	Age, date of nail return, smoking, age at first birth, parity, history of BBD, history of breast cancer in mother, history of breast cancer in a sister, age at menarche, menopausal status, BMI, alcohol consumption	8
Cui et al. 2007 [70], USA	Nested case-control (Kaiser Permanente Northwest cohort of women diagnosed with BBD in 1970–1994)	All women (252/252)	18–85 y (50% of cases and 55% of controls postmenopausal) at baseline (BBD diagnosis)	Benign breast tissue; X-ray fluorescence spectroscopy	Breast tissue iron (ng/cm^2^, normalized by sulfur content): highest (NR) vs. lowest (NR) quintile	All: 1.58 (1.02–2.44) Post: 2.77 (1.25–6.13)	No (only study that reported this measure)	Age, age at BBD diagnosis, duration of Kaiser Permanente membership, age at menarche, parity, age at first live birth, history of bilateral oophorectomy, family history of breast cancer, BMI, smoking, menopausal status, OC use, HRT, presence of proliferative changes in benign breast tissue	9
Moore et al. 2009 [61], China	Nested case-control (Breast Self Examination Trial cohort, 1989–2000)	All women (248/1040)	30–63 y at cohort enrolment; 35% postmenopausal at diagnosis	Plasma; immunoradiometric assay	Plasma ferritin (μg/L): highest (> 101.9) vs. lowest (≤18.9) quartile	All: 1.77 (0.96–3.27)	Yes	Age, year of blood draw	5
Stevens et al. 2011 [71], Japan	Nested case-control (Adult Health Study cohort, from the Life Span Study of atomic bomb survivors, 1969–2001); mean 13 y (range: 2 to 26 y)	All women (107/212) Premenopausal (15/29) Postmenopausal (92/183)	Age not specified; 86% postmenopausal at diagnosis (60% with postmenopausal serum)	Serum; chemiluminescent enzyme immunoassay	Serum ferritin (μg/L): per log unit increase	All: 1.3 (1.0–1.7) Pre: 1.0 (0.5–1.9) Post: 1.4 (1.1–1.9)	No (use tertile data below)	Matched by age at time of blood collection, menopausal status, sample collection year, and city; adjusted for radiation dose	7
					Premenopausal serum ferritin (log [μg/L]): highest (> 3.5 [> 33]) vs. lowest (< 2.4 [< 11]) tertile	Post: 1.1 (0.4–3.5)	Yes		
					Postmenopausal serum ferritin (log [μg/L]): highest (> 4.4 [> 81]) vs. lowest (< 3.8 [< 45]) tertile	Post: 2.5 (1.1–5.7)	Yes		
Gaur et al. 2013 [72], Sweden	Prospective cohort (Apolipoprotein Mortality Risk [AMORIS] Study, 1985–2002); mean 10.57 y	All women (3238/105795) Premenopausal cases: 1108 Postmenopausal cases: 2130	≥20 y at baseline	Serum; colorimetric assay on Technicon DAX 96 multichannel analyzer	Serum iron (μmol/L): highest (≥22) vs. lowest (< 14) quartile	Pre: 1.00 (0.83–1.20) Post: 1.24 (1.05–1.47)	Yes	Age, socioeconomic status, history of lung disease, CRP; model for serum iron also adjusted for TIBC and vice versa	8
					Serum TIBC (μmol/L): highest (≥67) vs. lowest (< 42) quartile	Pre: 1.06 (0.87–1.29) Post: 1.13 (0.96–1.33)	Yes		
Graff et al. 2014 [73], USA	Nested case-control (Nurses’ Health Study II, 1996–2009); mean 6.1 y (range: 1 month to 13.3 y)	All women (795/795) Premenopausal (406/402) Postmenopausal (299/301)	32–54 y and 24% postmenopausal at baseline (blood draw)	Plasma; electrochemi-luminescence immunoassay	Plasma ferritin (μg/L): highest (> 72.0) vs. lowest (≤23.9) quartile	All: 1.05 (0.77–1.45) Pre: 1.21 (0.77–1.88) Post: 1.09 (0.65–1.83) ER+/PR+: 1.14 (0.77–1.69) ER−/PR–: 0.70 (0.35–1.39)	Yes	Age at blood draw, race, menopausal status at blood draw and diagnosis, month/year of blood draw, luteal day at blood draw, time of day at blood draw, fasting status at blood draw, age at menarche, BMI at age 18, weight change since age 18, parity and age at first birth, family history of breast cancer, history of BBD	9
Wen et al. 2014 [41], Taiwan	Prospective cohort (MJ Health screening cohort, 1997–2008); median 7.07 y	All women (913/164355)	≥20 y at baseline	Serum; colorimetric assay on Abbott Architect C8000 automatic analyzer	Serum iron (μg/dL): highest (≥140) vs. referent (60–79) category; highest (≥140) vs. lowest (< 60) category	All: 1.31 (1.01–1.70); 1.62 (1.22–2.14)^c^	Yes	Age, BMI, systolic blood pressure, total cholesterol, CRP, hemoglobin, smoking, alcohol drinking, physical activity	7
Chua et al. 2016 [74], Australia	Prospective cohort (Busselton Health Survey, 1994–2010); 15–16 y	All women (80/1795) Premenopausal (39/775) Postmenopausal (41/1020)	25–79 y and 57% postmenopausal at baseline	Serum; chemiluminescence immunoassay (ferritin), colorimetric assay (iron), and immunoturbidimetry (transferrin) on an automated analyser	Serum ferritin (μg/L): highest (> 103) vs. lowest (< 53) tertile	All: 0.97 (0.54–1.74) Pre: 0.44 (0.16–1.23) Post: 1.74 (0.64–4.72)	Yes	Age, smoking, alcohol consumption, BMI, waist circumference, systolic blood pressure, diastolic blood pressure, high-density lipoprotein, triglycerides, glucose, HOMA-IR, CRP, alanine transaminase, γ-glutamyltransferase, bilirubin, albumin, menopausal status	9
					Serum iron (μmol/L): highest (≥20) vs. lowest (< 16) tertile	All: 1.64 (0.90–2.98) Pre: 1.14 (0.48–2.70) Post: 2.16 (0.91–5.12)	Yes		
					Serum TSAT (%): highest (≥30) vs. lowest (< 23) tertile	All: 1.90 (1.06–3.38) Pre: 1.27 (0.54–2.98) Post: 2.45 (1.08–5.58)	Yes		
Quintana Pacheco et al. 2018 [75], Germany	Case-cohort (European Prospective Investigation into Cancer and Nutrition [EPIC]-Heidelberg Study, 1994–2009); mean 15.7 y in the subcohort (8.4 y among breast cancer cases)	All women (627/1466)	35–65 y and 40% postmenopausal at baseline	Serum; Roche Cobas 6000 analytical system	Serum ferritin (μg/L): highest (median 193) vs. lowest (median 22) quartile	All: 0.67 (0.49–0.92) Pre: 0.66 (0.41–1.05) Post: 0.90 (0.53–1.54)	Yes	Age, waist circumference, height, alcohol consumption, CRP, smoking status, education, menopausal status, physical activity, current aspirin use, fibre intake, total red meat intake, energy intake, HRT use, current OC use, parity	9
					Serum iron (μmol/L): highest (median 25) vs. lowest (median 11) quartile	All: 1.04 (0.78–1.40)	Yes		
					Serum transferrin (μmol/L): highest (median 41) vs. lowest (median 29) quartile	All: 0.92 (0.70–1.23)	No (only study that reported this measure)		
					Serum TSAT (%): highest (median 53.8) vs. lowest (median 22.4) quartile	All: 1.03 (0.77–1.39)	Yes		

*Abbreviations*: *BBD* Benign breast disease, *BMI* Body mass index, *CRP* C-reactive protein, *CI* Confidence interval, *ER* Estrogen receptor, *FFQ* Food frequency questionnaire, *HRT* Hormone replacement therapy, *HOMA-IR* Homeostatic model assessment of insulin resistance, *NOS* Newcastle-Ottawa Scale, *NR* Not reported, *OC* Oral contraceptive, *Post* Postmenopausal, *Pre* Premenopausal, *PR* Progesterone receptor, *RR* Relative risk, *SD* Standard deviation, *TIBC* Total iron-binding capacity, *TSAT* Transferrin saturation

^a^ Cohort studies: number of cases/total number of participants; case-control studies: number of cases/number of controls

^b^ Results presented for pre- and postmenopausal breast cancer combined (All), premenopausal breast cancer (Pre), and/or postmenopausal breast cancer (Post), as well as by ER/PR status, where available

^c^ Calculated using tabulated raw data or RRs reported for other comparisons in the study

Of the 17 studies assessing iron intake, seven were cohort studies [39, 59, 62–66], with study size ranging from 4646 to 193,742 participants, follow-up ranging from 5.5 to 20 years, and number of breast cancer cases ranging from 188 to 9305; the remaining ten studies were case-control studies, of which four were hospital-based [53–56], three were population-based [42, 52, 60], and three were nested within existing cohorts [57, 58, 61], with case numbers ranging from 220 to 3452. Of the 11 studies assessing body iron status, five were cohort studies [41, 67, 68, 72, 74], with study size ranging from 1795 to 164,355 participants, follow-up ranging from 7.1 to 17.6 years, and number of cases ranging from 80 to 3238; the remaining six studies used a nested case-control [61, 69–71, 73] or case-cohort [75] design, with follow-up (where reported) ranging from 4 to 15.7 years and case numbers ranging from 107 to 795. Most studies consisted of both pre- and postmenopausal women across a wide age range at baseline and/or time of diagnosis, except for four cohort/nested case-control studies conducted among postmenopausal women alone [39, 58, 62, 66], one nested case-control study with primarily (86%) postmenopausal breast cancer cases [71], and one cohort study restricted to women who were premenopausal at baseline [63]. With respect to outcome ascertainment, incident breast cancer cases in prospective studies (e.g., cohort, nested case-control) were identified either through record linkage to cancer registries (and vital statistics) or through self-reports verified by medical records. Similarly, traditional case-control studies identified newly diagnosed cases (typically within 1 year of diagnosis, where reported) from cancer registries and/or hospital records.

With the exception of one cohort study where multiple 24-h dietary recalls were completed during the first 2 years of follow-up [65], all iron intake studies used a one-time, self- or interviewer-administered food frequency questionnaire (FFQ) to assess usual intake at baseline (cohort studies) or during a specified period (e.g., 2 years) before breast cancer diagnosis (case-control studies). Two studies involved more distant recall, including one assessing total and heme iron intake during adolescence [64] and one assessing dietary iron intake during preschool age [57], with FFQs completed retrospectively by participants at 33–52 years of age (start of follow-up) or by mothers of participants (after case diagnosis), respectively. Although the use of a previously validated (or pilot-tested [52]) FFQ was noted in all iron intake studies (Table 1), the validity and reproducibility of the FFQ have not always been directly assessed among the population under study. For example, several studies utilized an FFQ adapted from one that was designed and validated for a different study without re-evaluating its performance in the current study population [42, 54–57, 61].

Of the 11 studies examining iron status, nine assessed one or more serum/plasma biomarkers (ferritin, iron, transferrin, TIBC, and/or TSAT) [41, 61, 67, 68, 71–75], while the other two analyzed iron levels in toenail [69] or benign breast tissue [70]. Two of these studies noted that biological samples were collected at more than one time point for a small proportion of participants, including a cohort study where 23% of women provided serum samples at two or three health examinations [68] and a nested case-control study where 5% of cases and 2% of controls had both pre- and postmenopausal serum samples [71]. It can be assumed that all other studies involved measurements taken at a single time point (i.e., baseline).

Results of almost all included studies were reported as RRs across quantiles (tertiles, quartiles, or quintiles) of iron intake or status. While age was matched and/or adjusted for in all studies, the level of adjustment of other potential confounders differed across studies (Table 1). Most iron intake studies included total energy intake as a covariate in the multivariable model, regardless of whether the iron intake variable (other than supplemental iron) itself was crude (i.e., absolute intake) [42, 54–56, 60, 61, 65] or adjusted for energy using the nutrient density [39, 62, 66] or the residual [39, 53, 57–59, 63, 64] method. Other commonly adjusted variables in iron intake studies included BMI, alcohol intake, family history of breast cancer, and reproductive/hormonal factors, such as age at menarche, parity, age at menopause, and OC and/or HRT use. Several studies also adjusted for education, smoking, physical activity, history of benign breast disease (BBD), and/or dietary factors (e.g., fat intake). Iron status studies, especially those where breast cancer was not the only outcome of interest, generally had more limited adjustment for established breast cancer risk factors (e.g., reproductive history). Notably, four recent iron status studies adjusted for C-reactive protein (CRP) as a marker of inflammation [41, 72, 74, 75].

Details of the quality assessment of individual studies are presented in Additional file 3: Table S1. Overall, NOS scores ranged from 4 to 9 (mean: 7.0). For the 17 studies examining iron intake, scores ranged from 4 to 8 (mean: 6.5), with 10 studies considered to be of high-quality (NOS ≥7) [39, 52, 58–60, 62–66] and seven studies to be of low-quality (NOS < 7) [42, 53–57, 61]. For the 11 studies examining iron status, NOS scores ranged from 5 to 9 (mean: 7.7), with only one study scoring below 7 [61].

### Iron intake and breast cancer risk

#### Highest vs. lowest analysis

**Fig. 2:**
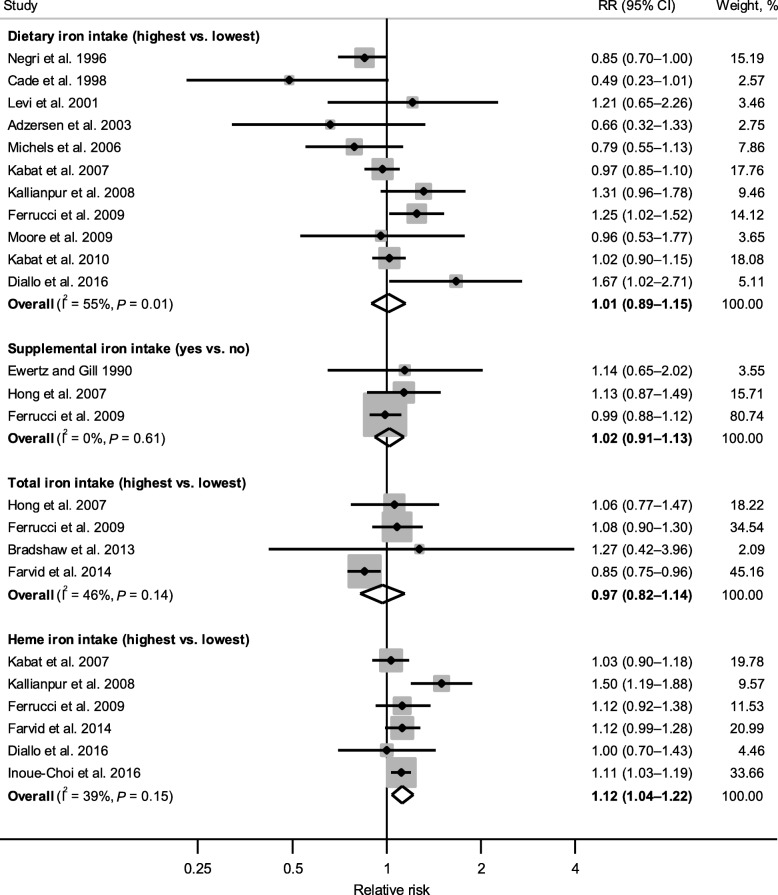
Forest plot of associations between iron intake (highest vs. lowest category) and breast cancer risk. The diamonds represent the pooled relative risks and corresponding 95% confidence intervals obtained from random-effects meta-analyses. The dots and horizontal lines represent the relative risks and corresponding 95% confidence intervals of individual studies, and the sizes of shaded squares are proportional to the weight contributed by each study to the pooled estimate. I^2^ is the proportion of the total variability attributable to between-study heterogeneity, and *P* is from Cochran’s Q test evaluating the presence of heterogeneity

Figure 2 shows forest plots for the associations of dietary, supplemental, total, and heme iron intake (yes vs. no for supplemental iron; highest vs. lowest intake category for all other measures) with breast cancer risk.

A meta-analysis combining estimates from 11 studies [39, 53–57, 59–62, 65] did not reveal an association between dietary iron intake and breast cancer risk, with a pooled RR of 1.01 (95% CI: 0.89–1.15); however, significant heterogeneity was detected across studies (I^2^ = 55%, *P*_heterogeneity_ = 0.01). With the exception of the one study reporting intake during preschool age (highest vs. lowest quintile, mean intakes of 7.23 and 2.54 mg/day, respectively) [57], dietary iron intake levels across studies (where reported) ranged between > 11.9 and > 17.5 mg/day for the highest category and between < 9.0 and ≤ 12.0 mg/day for the lowest (referent) category. Results did not change appreciably when the study assessing preschool iron intake [57] was excluded from the analysis (pooled RR = 1.04, 95% CI: 0.91–1.18; I^2^ = 56%, *P*_heterogeneity_ = 0.02).

Similarly, no associations were found for intakes of supplemental iron (pooled RR = 1.02, 95% CI: 0.91–1.13; I^2^ = 0%, *P*_heterogeneity_ = 0.61) and total iron (pooled RR = 0.97, 95% CI: 0.82–1.14; I^2^ = 46%, *P*_heterogeneity_ = 0.14), based on results combined from three studies [39, 52, 58] and four studies [39, 42, 58, 63], respectively.

In contrast, heme iron intake showed a significant positive association with breast cancer risk based on six studies [39, 59, 60, 63, 65, 66], with a pooled RR of 1.12 (95% CI: 1.04–1.22) and low-to-moderate heterogeneity (I^2^ = 39%, *P*_heterogeneity_ = 0.15). This association persisted after excluding the two studies where animal [60] or red meat [65] derived iron was used as a proxy measure for heme iron intake (pooled RR = 1.10, 95% CI: 1.04–1.16; I^2^ = 0%, *P*_heterogeneity_ = 0.78), or when restricting to studies that used a previously developed laboratory-based database to assess heme iron intake [39, 66] (pooled RR = 1.11, 95% CI: 1.04–1.19; I^2^ = 0%, *P*_heterogeneity_ = 0.94). A meta-analysis was not conducted for non-heme iron intake, as only one study reported its association (assessed as plant-derived iron) with breast cancer risk (RR [highest vs. lowest quartile] = 0.99, 95% CI: 0.75–1.29) [60].

Table 2 presents results from subgroup analyses for dietary, total, and heme iron intake (not conducted for supplemental iron due to limited number of studies). The association between dietary iron intake and breast cancer risk did not differ significantly among subgroups defined by study design, geographic location, menopausal status, dietary assessment method, or adjustments for specific confounders (*P*_difference_ > 0.10 from meta-regression), with substantial heterogeneity remaining within most subgroups. However, when stratified by study quality, a significant inverse association was observed for low-quality studies (pooled RR = 0.84, 95% CI: 0.72–0.96), whereas a positive but nonsignificant association was seen for high-quality studies (pooled RR = 1.12, 95% CI: 0.98–1.29) (*P*_difference_ = 0.03), suggesting study quality may be a contributor to heterogeneity. Furthermore, post-hoc subgroup analyses stratifying results by the highest dietary iron intake category (> 15 mg/day, ≤15 mg/day, or not reported) and method of energy adjustment (covariate only, nutrient density, or residual method) did not reveal significant differences (*P*_difference_ = 0.96 and 0.32, respectively; data not shown). No notable differences were observed for total iron intake, which remained unassociated with breast cancer risk across subgroups. For heme iron intake, all six studies were of high-quality, and significant positive associations remained among cohort studies and studies conducted in North America. In addition, heme iron intake showed a slightly stronger association with premenopausal (pooled RR = 1.21, 95% CI: 0.97–1.51) than postmenopausal (pooled RR = 1.08, 95% CI: 0.99–1.18) breast cancer, although statistical significance was not reached in either subgroup.

**Table 2:** Subgroup analyses for the associations of dietary, total, and heme iron intake with breast cancer risk

Subgroups	Dietary iron intake (highest vs. lowest)					Total iron intake (highest vs. lowest)				Heme iron intake (highest vs. lowest)			
	No. of RRs	RR (95% CI)	I^2^ (%)^a^	*P*_heterogeneity_^b^	*P*_difference_^c^	No. of RRs	RR (95% CI)	I^2^ (%)^a^	*P*_heterogeneity_^b^	No. of RRs	RR (95% CI)	I^2^ (%)^a^	*P*_heterogeneity_^b^
Overall	11	1.01 (0.89–1.15)	55	0.01		4	0.97 (0.82–1.14)	46	0.14	6	1.12 (1.04–1.22)	39	0.15
Study design					0.20								
Cohort	4	1.10 (0.94–1.27)	63	0.05		2	0.95 (0.75–1.20)	78	0.03	5	1.10 (1.04–1.16)	0	0.86
Case-control	7	0.91 (0.73–1.12)	45	0.10		2	1.08 (0.79–1.47)	0	0.76	1	1.50 (1.19–1.88)	NA	NA
Geographic location					0.65								
North America	4	1.02 (0.90–1.16)	54	0.09		4	0.97 (0.82–1.14)	46	0.14	4	1.10 (1.04–1.16)	0	0.78
Europe	5	0.93 (0.65–1.34)	63	0.03		0	NA	NA	NA	1	1.00 (0.70–1.43)	NA	NA
Asia	2	1.23 (0.93–1.62)	0	0.37		0	NA	NA	NA	1	1.50 (1.19–1.88)	NA	NA
Menopausal status^d^					0.78								
Premenopausal	3	1.12 (0.94–1.32)	0	0.62		1	0.88 (0.74–1.04)	NA	NA	3	1.21 (0.97–1.51)	68	0.05
Postmenopausal	5	1.11 (0.92–1.33)	64	0.03		3	0.97 (0.81–1.17)	50	0.14	5	1.08 (0.99–1.18)	21	0.28
Study quality					0.03								
High (NOS score ≥ 7)	5	1.12 (0.98–1.29)	60	0.04		3	0.97 (0.81–1.16)	62	0.07	6	1.12 (1.04–1.22)	39	0.15
Low (NOS score < 7)	6	0.84 (0.72–0.96)	0	0.53		1	1.27 (0.41–3.92)	NA	NA	0	NA	NA	NA
Dietary assessment method					0.34								
Structured interview	5	1.13 (0.85–1.51)	63	0.03		0	NA	NA	NA	2	1.25 (0.85–1.86)	72	0.06
Self-administered	6	0.98 (0.84–1.13)	57	0.04		4	0.97 (0.82–1.14)	46	0.14	4	1.10 (1.04–1.16)	0	0.78
Adjustments for confounders													
BMI					0.33								
Yes	9	1.05 (0.91–1.21)	56	0.02		2	0.95 (0.75–1.20)	78	0.03	6	1.12 (1.04–1.22)	39	0.15
No	2	0.86 (0.72–1.02)	0	0.70		2	1.08 (0.79–1.47)	0	0.76	0	NA	NA	NA
Physical activity					0.13								
Yes	3	1.22 (0.93–1.59)	63	0.07		0	NA	NA	NA	3	1.20 (0.96–1.48)	70	0.04
No	8	0.94 (0.80–1.10)	52	0.04		4	0.97 (0.82–1.14)	46	0.14	3	1.08 (1.00–1.18)	0	0.64
Alcohol intake					0.95								
Yes	8	1.01 (0.88–1.17)	61	0.01		2	0.95 (0.75–1.20)	78	0.03	5	1.10 (1.04–1.16)	0	0.86
No	3	1.02 (0.72–1.44)	55	0.11		2	1.08 (0.79–1.47)	0	0.76	1	1.50 (1.19–1.88)	NA	NA
OC and/or HRT use					0.35								
Yes	5	1.08 (0.93–1.25)	59	0.05		2	0.95 (0.75–1.20)	78	0.03	5	1.10 (1.04–1.16)	0	0.86
No	6	0.93 (0.74–1.17)	50	0.08		2	1.08 (0.79–1.47)	0	0.76	1	1.50 (1.19–1.88)	NA	NA
Family history of breast cancer					0.20								
Yes	7	1.07 (0.93–1.23)	57	0.03		2	0.95 (0.75–1.20)	78	0.03	6	1.12 (1.04–1.22)	39	0.15
No	4	0.86 (0.69–1.07)	15	0.32		2	1.08 (0.79–1.47)	0	0.76	0	NA	NA	NA

*Abbreviations*: *BMI* Body mass index, *CI* Confidence interval, *HRT* Hormone replacement therapy, *NA* Not applicable, *NOS* Newcastle-Ottawa Scale, *OC* Oral contraceptive, *RR* Relative risk

^a^ I^2^ statistics indicating the proportion of the total variability attributable to between-study heterogeneity

^b^ *P* values from Cochran’s Q test evaluating the presence of heterogeneity across studies

^c^ *P* values for difference between subgroups calculated from meta-regression, conducted only for dietary iron intake (i.e., at least 10 studies available)

^d^ Pooled estimates were calculated only from studies providing menopausal status-specific results

#### Dose-response analysis

**Fig. 3:**
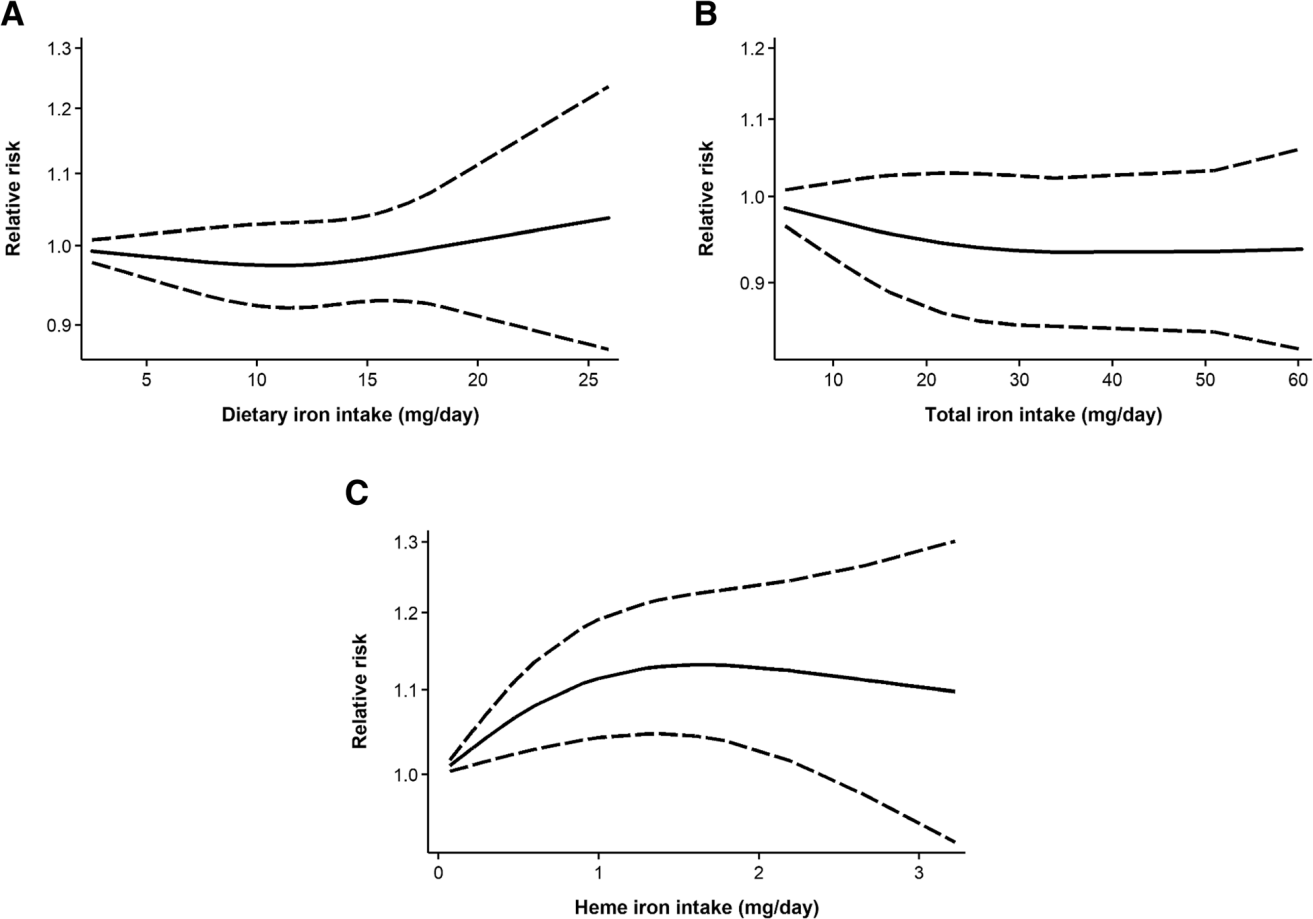
Dose-response curves for intakes of (**a**) dietary iron; (**b**) total iron; and (**c**) heme iron in relation to breast cancer risk. Data were modeled using random-effects restricted cubic spline models with three knots fixed at the 10th, 50th, and 90th percentiles. The solid lines represent the fitted relative risks for the nonlinear trend, and the dashed lines represent pointwise 95% confidence intervals

Similar to the highest vs. lowest analysis, linear dose-response meta-analyses (Additional file 4: Figure S1) revealed no associations between either dietary or total iron intake and breast cancer risk, with pooled RRs of 1.00 (95% CI: 0.97–1.03) and 1.00 (95% CI: 0.98–1.01), respectively, per 5-mg/day increase in intake. In contrast, each 1-mg/day increase in heme iron intake, was associated with a statistically significant 8% increase in breast cancer risk (pooled RR = 1.08, 95% CI: 1.002–1.17). Based on nonlinear dose-response meta-analyses, no significant curvilinear associations with breast cancer risk were found for intakes of dietary iron (*P*_nonlinearity_ = 0.41; Fig. 3a) and total iron (*P*_nonlinearity_ = 0.46; Fig. 3b), although a small decrease in risk nearing statistical significance was observed across levels of total iron. Meanwhile, there appeared to be a threshold effect in the dose-response curve for heme iron intake (*P*_nonlinearity_ = 0.03; Fig. 3c), with risk leveling off at approximately 1 mg/day.

### Body iron status and breast cancer risk

#### Highest vs. lowest analysis

**Fig. 4:**
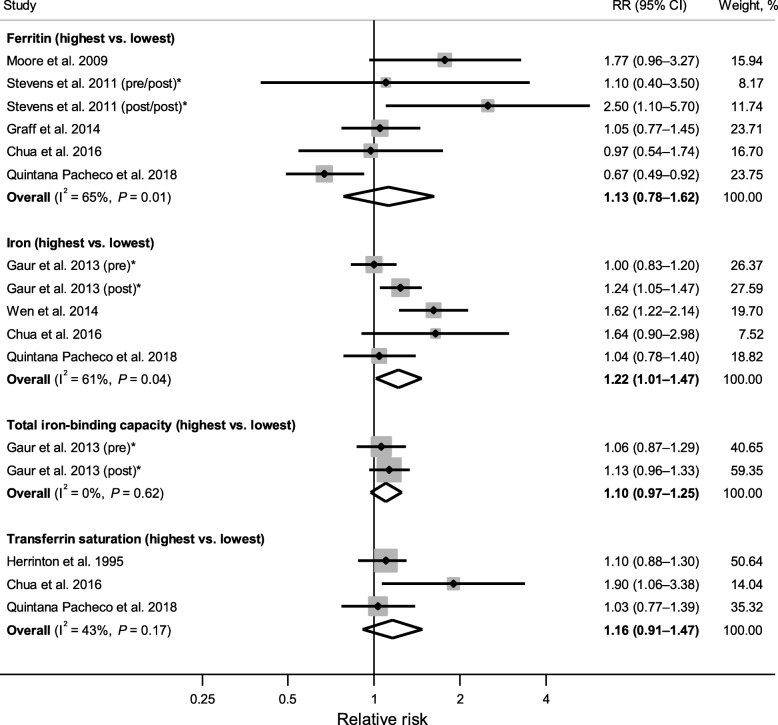
Forest plot of associations between serum/plasma indicators of body iron status (highest vs. lowest category) and breast cancer risk. The diamonds represent the pooled relative risks and corresponding 95% confidence intervals obtained from random-effects meta-analyses. The dots and horizontal lines represent the relative risks and corresponding 95% confidence intervals of individual studies, and the sizes of shaded squares are proportional to the weight contributed by each study to the pooled estimate. I^2^ is the proportion of the total variability attributable to between-study heterogeneity, and *P* is from Cochran’s Q test evaluating the presence of heterogeneity. *Stevens et al. 2011 [71] reported separate estimates for premenopausal (pre/post) and postmenopausal (post/post) ferritin levels in relation to postmenopausal breast cancer risk; Gaur et al. 2013 [72] reported separate estimates for premenopausal (pre) and postmenopausal (post) breast cancer

Figure 4 shows forest plots of associations between each serum/plasma indicator of body iron status (highest vs. lowest category) and breast cancer risk. A meta-analysis combining five RRs derived from four studies [41, 72, 74, 75] (one study reported separate RRs for pre- and postmenopausal breast cancer [72]) revealed a significant positive association between serum iron and breast cancer risk (pooled RR = 1.22, 95% CI: 1.01–1.47), with significant heterogeneity (I^2^ = 61%, *P*_heterogeneity_ = 0.04). The associations were also in the positive direction, but not statistically significant, for ferritin (pooled RR = 1.13, 95% CI: 0.78–1.62; I^2^ = 65%, *P*_heterogeneity_ = 0.01) and TSAT (pooled RR = 1.16, 95% CI: 0.91–1.47; I^2^ = 43%, *P*_heterogeneity_ = 0.17), based on six RRs from five studies [61, 71, 73–75] and three RRs from three studies [68, 74, 75], respectively. High levels of TIBC, which is indicative of low body iron status, was not associated with breast cancer risk when two RRs from one study [72] were combined (pooled RR = 1.10, 95% CI: 0.97–1.25; I^2^ = 0%, *P*_heterogeneity_ = 0.62). Similarly, serum transferrin was not associated with breast cancer risk according to the only study reporting this measure (RR = 0.92, 95% CI: 0.70–1.23) [75]. Meta-analysis combining results for transferrin and TIBC (proxy measure of transferrin) also revealed no significant association (pooled RR = 1.07, 95% CI: 0.95–1.20; I^2^ = 0%, *P*_heterogeneity_ = 0.46; data not shown).

Table 3 presents results from subgroup analyses for serum/plasma ferritin and iron. Despite the lack of association overall, ferritin was significantly associated with increased breast cancer risk among studies conducted in Asia (pooled RR = 1.81, 95% CI: 1.16–2.83). In general, pooled RRs from ferritin studies adjusting for potential confounders (e.g., BMI, physical activity, alcohol intake) showed nonsignificant inverse associations, whereas those not adjusting for confounders showed positive associations; slightly stronger positive associations were seen among iron studies adjusting for confounders. For both ferritin and iron, a stronger positive association was observed for postmenopausal (ferritin: pooled RR = 1.23, 95% CI: 0.87–1.75; iron: pooled RR = 1.39, 95% CI: 0.90–2.15) than premenopausal (ferritin: pooled RR = 0.79, 95% CI: 0.46–1.35; iron: pooled RR = 1.01, 95% CI: 0.84–1.20) breast cancer; however, statistical significance was not reached within subgroups.

**Table 3:** Subgroup analyses for the associations of serum/plasma ferritin and iron with breast cancer risk

Subgroups	Serum/plasma ferritin (highest vs. lowest)				Serum/plasma iron (highest vs. lowest)			
	No. of RRs	RR (95% CI)	I^2^ (%)^a^	*P*_heterogeneity_^b^	No. of RRs	RR (95% CI)	I^2^ (%)^a^	*P*_heterogeneity_^b^
Overall	6	1.13 (0.78–1.62)	65	0.01	5	1.22 (1.01–1.47)	61	0.04
Study design								
Cohort	1	0.97 (0.54–1.74)	NA	NA	4	1.27 (1.02–1.59)	68	0.03
Nested case-control/case-cohort	5	1.18 (0.76–1.84)	72	0.01	1	1.04 (0.78–1.40)	NA	NA
Geographic location								
North America	1	1.05 (0.77–1.45)	NA	NA	0	NA	NA	NA
Europe	1	0.67 (0.49–0.92)	NA	NA	3	1.10 (0.95–1.28)	35	0.21
Asia	3	1.81 (1.16–2.83)	0	0.50	1	1.62 (1.22–2.14)	NA	NA
Australia	1	0.97 (0.54–1.74)	NA	NA	1	1.64 (0.90–2.98)	NA	NA
Menopausal status^c^								
Premenopausal	3	0.79 (0.46–1.35)	61	0.08	2	1.01 (0.84–1.20)	0	0.78
Postmenopausal	5	1.23 (0.87–1.75)	18	0.30	2	1.39 (0.90–2.15)	35	0.21
Study quality								
High (NOS score ≥ 7)	5	1.02 (0.70–1.48)	61	0.04	5	1.22 (1.01–1.47)	61	0.04
Low (NOS score < 7)	1	1.77 (0.96–3.27)	NA	NA	0	NA	NA	NA
Biological sample								
Serum	4	1.06 (0.61–1.85)	67	0.03	5	1.22 (1.01–1.47)	61	0.04
Plasma	2	1.27 (0.78–2.09)	55	0.14	0	NA	NA	NA
Adjustments for confounders								
BMI								
Yes	3	0.86 (0.63–1.19)	51	0.13	3	1.36 (0.98–1.90)	61	0.08
No	3	1.81 (1.16–2.83)	0	0.50	2	1.12 (0.91–1.38)	65	0.09
Physical activity								
Yes	1	0.67 (0.49–0.92)	NA	NA	2	1.30 (0.84–2.00)	78	0.03
No	5	1.28 (0.93–1.76)	31	0.21	3	1.16 (0.94–1.43)	54	0.12
Alcohol intake								
Yes	2	0.74 (0.54–1.03)	18	0.27	3	1.36 (0.98–1.90)	61	0.08
No	4	1.41 (0.93–2.13)	42	0.16	2	1.12 (0.91–1.38)	65	0.09
OC and/or HRT use								
Yes	1	0.67 (0.49–0.92)	NA	NA	1	1.04 (0.78–1.40)	NA	NA
No	5	1.28 (0.93–1.76)	31	0.21	4	1.27 (1.02–1.59)	68	0.03
Family history of breast cancer								
Yes	1	1.05 (0.77–1.45)	NA	NA	0	NA	NA	NA
No	5	1.19 (0.71–2.00)	72	0.01	5	1.22 (1.01–1.47)	61	0.04

*Abbreviations*: *BMI* Body mass index, *CI* Confidence interval, *HRT* Hormone replacement therapy, *NA* Not applicable, *NOS* Newcastle-Ottawa Scale, *OC* Oral contraceptive, *RR* Relative risk

^a^ I^2^ statistics indicating the proportion of the total variability attributable to between-study heterogeneity

^b^ *P* values from Cochran’s Q test evaluating the presence of heterogeneity across studies

^c^ Pooled estimates were calculated only from studies providing menopausal status-specific results

#### Dose-response analysis

**Fig. 5:**
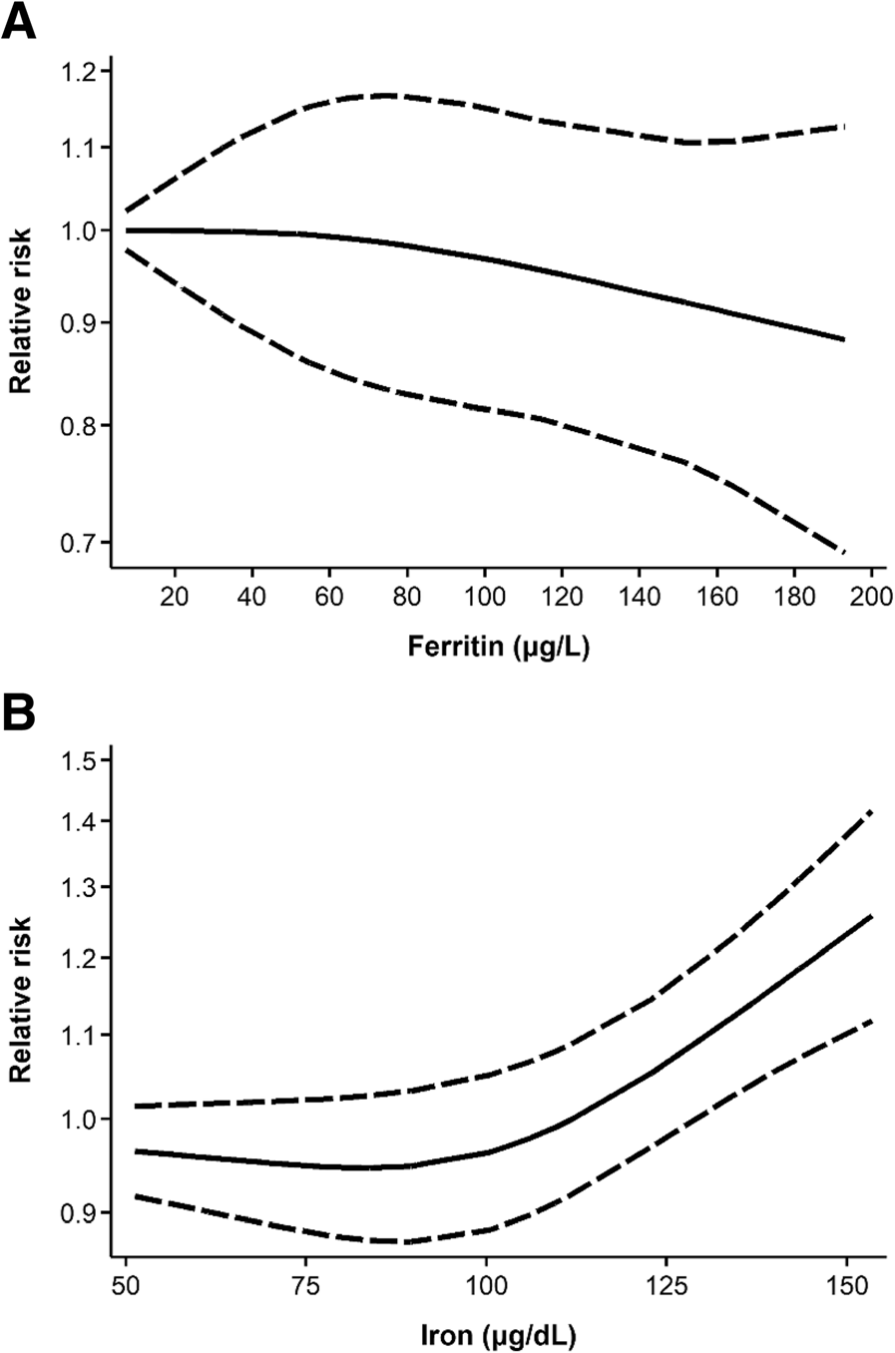
Dose-response curves for serum/plasma (**a**) ferritin and (**b**) iron in relation to breast cancer risk. Data were modeled using random-effects restricted cubic spline models with three knots fixed at the 10th, 50th, and 90th percentiles. The solid lines represent the fitted relative risks for the nonlinear trend, and the dashed lines represent pointwise 95% confidence intervals

No significant linear associations were found between any of the serum/plasma indicators of body iron status and breast cancer risk (Additional file 4: Figure S2). The dose-response curve for ferritin suggested a tendency towards a decrease in breast cancer risk with increasing concentration; however, the CIs were wide due to heterogeneous results and included the null value across all ferritin levels, and no departure from linearity was detected (*P*_nonlinearity_ = 0.70) (Fig. 5a). On the other hand, serum iron exhibited a J-shaped dose-response relationship with breast cancer risk, with strong evidence of a nonlinear effect (*P*_nonlinearity_ < 0.001) (Fig. 5b). Specifically, a steady increase in risk was noted for serum iron levels above ~ 100 μg/dL, with the association becoming statistically significant at just beyond ~ 125 μg/dL. No evidence of curvilinear associations was found for TIBC or TSAT (data not shown).

#### Other iron biomarkers

Two nested case-control studies assessed iron biomarkers in samples other than serum or plasma [69, 70]. In the only study assessing toenail iron in relation to breast cancer risk, no overall association was observed (RR [highest vs. lowest quintile] = 0.89, 95% CI: 0.56–1.40) [69]. However, when stratified by menopausal status, toenail iron was inversely associated with premenopausal (RR [highest vs. lowest quintile] = 0.45, 95% CI: 0.21–0.95) and positively associated with postmenopausal (RR [highest vs. lowest quintile] = 1.56, 95% CI: 0.80–3.03) breast cancer (*P*_interaction_ = 0.08). In another study where iron levels were measured in benign breast tissue among women with BBD, an elevated breast cancer risk was observed overall (RR [highest vs. lowest quintile] = 1.58, 95% CI: 1.02–2.44) and in postmenopausal women (RR [highest vs. lowest quintile] = 2.77, 95% CI: 1.25–6.13) [70].

### Publication bias and sensitivity analysis

No publication bias was detected in the highest vs. lowest meta-analyses for dietary iron (Begg’s *P* = 0.48; Egger’s *P* = 0.91), supplemental iron (Begg’s *P* = 0.60; Egger’s *P* = 0.35), total iron (Begg’s *P* > 0.99; Egger’s *P* = 0.39), and heme iron (Begg’s *P* = 0.85; Egger’s *P* = 0.64) intake, or for serum/plasma ferritin (Begg’s *P* = 0.19; Egger’s *P* = 0.17), iron (Begg’s *P* = 0.62; Egger’s *P* = 0.47), or TSAT (Begg’s *P* = 0.60; Egger’s *P* = 0.41), whereas publication bias was detected for the combined analysis of TIBC and transferrin (Begg’s *P* = 0.12; Egger’s *P* = 0.02). Visual inspection of the funnel plots (Additional file 5: Figures S3 and S4) indicated some asymmetry for total iron intake and serum/plasma indicators of iron status (i.e., ferritin, iron, and TSAT), where smaller studies with inverse associations may have been excluded; however, this was based only on a limited number of studies. There was no statistical evidence of publication bias in the dose-response meta-analyses (Begg’s and Egger’s *P* > 0.10 for all).

No notable changes in the pooled estimates were observed when individual studies were omitted one at time in the sensitivity analyses (Additional file 6: Figures S5–S8), although the association between the highest (vs. lowest) level of serum iron and breast cancer risk lost statistical significance in some cases given the small number of studies.

## Discussion

The results of our systematic review and meta-analysis suggest that heme iron intake is positively associated with breast cancer risk, with a statistically significant 12% increase in risk when comparing the highest vs. lowest level of intake and 8% increase in risk for each 1-mg/day increase in intake. In contrast, no associations were found for dietary, supplemental, total, or non-heme iron intake. Among serum/plasma indicators of body iron status, the highest (vs. lowest) level of iron, but not ferritin, transferrin, TIBC, or TSAT, also showed a statistically significant association with increased breast cancer risk (22%). Furthermore, dose-response meta-analyses indicated a nonlinear threshold effect for heme iron intake and a J-shaped pattern for serum iron in relation to breast cancer risk.

This is the first systematic review and meta-analysis specifically assessing breast cancer risk in relation to various measures of iron intake and body iron status. Our review identified many additional studies not included in the previous systematic review/meta-analysis on iron and cancer risk [29], which only identified seven studies assessing iron intake [39, 54–56, 59, 60, 62] and zero studies assessing body iron status (versus 17 and 11 studies, respectively, in our review), in relation to breast cancer risk. While this discrepancy is partly due to the narrower range of publication year (1995–2012) [29] compared to the current review (up to 2018), the use of only one electronic database (versus four databases plus manual search of reference lists in our review), as well as a limited set of relevant search terms (e.g., missing specific iron biomarker terms, such as “ferritin” and “transferrin”), may also explain the considerably smaller number of studies identified in the previous review.

In contrast to our finding of a positive association between heme iron intake and breast cancer risk, the previous meta-analysis reported a lack of association between heme iron intake and breast cancer risk (pooled RR [per 1-mg/day] = 1.03, 95% CI: 0.97–1.09) based on only three studies [29]. The inclusion of recent additional studies in our analysis, including larger cohort studies with longer follow-up [63, 65, 66], likely increased statistical power to detect the relatively modest association. Our results were, however, consistent with meta-analyses evaluating heme iron intake in relation to colorectal cancer risk [29, 76, 77]. The catalytic effects of heme iron on endogenous *N-*nitrosation and lipid peroxidation, and subsequent oxidative damage to cellular biomolecules, have been suggested to contribute to the development of both colorectal and breast cancer [76, 78]. Furthermore, differences in bioavailability may explain why an association with breast cancer risk was found only for heme, and not for non-heme (or overall dietary), iron intake [79]. Surrounded by a water-soluble porphyrin ring, heme iron is more efficiently absorbed by intestinal cells [79] and is a stronger predictor of body iron status [80–82] compared to non-heme iron. Heme iron absorption is also less influenced by the body’s iron requirements or the presence of other dietary components known to enhance (e.g., vitamin C) or inhibit (e.g., phytate) non-heme iron uptake [83].

Interestingly, heme iron intake exhibited a nonlinear threshold effect in our dose-response meta-analysis, although absolute intake values should be interpreted with caution given differences in methods used to assess heme iron levels across studies. For example, while literature-based meat-specific percentages (e.g., 69% in beef, 39% in pork/ham/luncheon meats, 26% in chicken and fish, 21% in liver) [13, 14] were applied in some studies [59, 63], others [39, 62, 66] used a laboratory-based heme iron database (restricted to certain meats) that accounts for meat type, cooking method, and doneness level [14]. Nevertheless, regardless of heme iron assessment method, this threshold effect was also evident in several individual studies where a significant association with breast cancer risk was observed in one or more of the middle heme iron intake quantiles and leveled off (or became weaker and lost statistical significance) in the highest quantile [39, 59, 62, 63]. Furthermore, since heme iron is derived only from animal source foods, with particularly high content in (and hence highly correlated with) red meat, the possibility that other red meat components (e.g., fat, meat mutagens) contributed to the association observed cannot be precluded [39, 84]. However, the association between red meat intake (and meat mutagens) and breast cancer risk is not strongly supported by current epidemiologic literature [39, 84–87], and most studies assessing heme iron in our review adjusted for fat intake as a potential confounder.

No overall association was observed between dietary iron intake and breast cancer risk, although results were heterogeneous, with studies reporting positive [39, 65], inverse [53, 54], or null [55–57, 59–62] associations. While subgroup and meta-regression analyses suggested that study quality may partly explain the heterogeneity, a closer examination of the lower-quality studies (where an inverse association was found) indicated that they were either hospital-based [53–56] or nested [57, 61] case-control studies with FFQs administered after breast cancer diagnosis or just before biopsy. Thus, differential reporting of dietary intake between cases and controls, as well as potential biases related to hospital-based control selection, may have contributed to the inverse association. Even among higher-quality studies (all cohort or population-based case-control) where the association was in the positive direction, substantial heterogeneity was present. Given the association observed between heme iron intake and breast cancer risk, the relative contribution of heme and non-heme iron to overall dietary iron intake among different study populations may be another possible source of heterogeneity. This also highlights the importance of considering different subtypes of dietary iron in future studies, especially since our review only identified one study assessing non-heme (plant-derived) iron in addition to heme iron intake [60].

Our meta-analysis provided no evidence of an association between supplemental or total iron intake and breast cancer risk; however, the dose-response curve for total iron intake (Fig. 3b) suggested a weakly protective effect nearing statistical significance. Residual confounding by health behaviours, which are likely associated with supplement use (and hence high levels of total iron intake), may have partly contributed to this inverse trend. Nevertheless, only a few studies examined these associations, and the assessment of supplemental iron was not always comprehensive (e.g., did not include both single-ingredient iron supplements and iron-containing multivitamin/mineral products) or clearly described. Although iron supplements typically contain non-heme iron (e.g., ferrous sulfate), they deliver high doses of iron that account for a major proportion of total iron intake among users [88]. Moreover, use of iron-containing supplements (both single and multi-ingredient products) is especially common among female populations [89, 90], suggesting that dietary iron alone would substantially underestimate total iron intake. Thus, additional studies with detailed assessments of supplemental (and total) iron intake, including dosage and frequency/duration of use, are warranted before reaching firm conclusions about their associations with breast cancer risk.

In contrast to the lack of association observed for total and dietary iron intake, high levels of serum iron were found to be associated with increased breast cancer risk. Similarly, although based only on single studies, iron measured in toenail [69] and benign breast tissue [70] may also exhibit a positive association with breast cancer risk, especially in postmenopausal women. Given limitations of dietary assessment, as well as inter-individual variation in iron absorption and metabolism, biomarkers may better reflect exposure to biologically available iron than dietary intake [91]. Thus, our results provide some support for the role of biologically available iron in breast cancer etiology. Conversely, the review by Fonseca-Nunes et al. suggested an inverse association between body iron status and gastrointestinal cancers [29], while several studies reported sex differences in the associations of iron biomarkers (e.g., positive for women and inverse or null for men) with overall cancer risk [74, 92]. It is possible that iron exerts different effects on different cancer sites and in women (vs. men), among whom iron-induced carcinogenesis likely involves a complex interplay with reproductive/hormonal factors [7, 93]. Furthermore, the J-shaped dose-response we observed between serum iron and breast cancer risk is similar to a study assessing serum iron in relation to overall cancer risk [41]. Individuals with very low body iron levels, such as those with iron-deficiency anemia, may be distinct from others (e.g., altered immune function) with respect to cancer risk [94], suggesting the need to consider these individuals as a separate group or to assess iron levels as a continuous variable without assuming a linear dose-response.

The significant positive association observed for serum iron but not ferritin in our meta-analysis has also been reported by one study examining multiple iron biomarkers within a single population [74], suggesting that circulating iron may be more relevant to breast carcinogenesis than stored iron (ferritin); however, this warrants additional investigation, given the significant heterogeneity detected for both ferritin and iron and the small number of studies assessing these measures. Serum iron has been suggested as a poorer indicator of iron status and is subject to greater within-person variability (30%) compared to ferritin (10–25%) [18]. In addition, serum biomarkers may not be reliable indicators of iron status in the presence of inflammation, where ferritin levels are elevated and iron and transferrin are decreased [95]. These limitations highlight the need to measure iron biomarkers at multiple time points, to explore use of novel or more stable indicators of iron status, and to evaluate the potential impact of inflammation on iron status measures in future studies.

Although stratified analyses by menopausal status generally revealed no significant associations due to limited statistical power, several indicators of iron status (serum/plasma ferritin and iron, toenail iron, and breast tissue iron) appeared to be more strongly associated with increased postmenopausal breast cancer risk. A possible explanation is age-related dysregulation of iron metabolism and declines in antioxidant defense mechanisms [96]. Conversely, the associations for iron intake did not differ by menopausal status, except for a slightly stronger association between heme iron intake and premenopausal breast cancer risk; this is unexpected since postmenopausal women, who are no longer losing iron through menstruation, are more likely to accumulate iron in their body [97]. Furthermore, it was surprising to find that only two studies (one assessing heme iron intake [66] and one assessing ferritin [73]) investigated associations according to tumour hormone receptor subtype. Although neither of these studies reported significant differences, additional studies assessing ER/PR status are warranted to explore potential etiologic heterogeneity, especially given in vitro evidence suggesting a stronger role of iron in ER-positive breast carcinogenesis [98].

This is the first systematic review and meta-analysis specifically assessing the associations between various measures of iron intake, as well as body iron status, and risk of breast cancer. A major strength is the extensive search strategy, allowing us to identify many additional studies not included in the 2014 review on iron and cancer risk [29], especially those evaluating body iron status in relation to breast cancer risk. Importantly, our review included detailed assessments of study quality and provided a comprehensive and quantitative synthesis of findings, including subgroup analyses to explore sources of heterogeneity. Furthermore, in addition to category-based (highest vs. lowest) analyses, we also performed dose-response meta-analyses to examine linear and nonlinear relationships.

Several limitations should be considered when interpreting the findings of this review. First, the meta-analysis for some iron measures was based only on a small number of studies, which could have resulted in limited statistical power for the overall or subgroup analyses and the assessments of publication bias, as well as greater influence of single studies. Nevertheless, sensitivity analyses with individual studies omitted one at a time generally led to no notable changes in the pooled estimates, indicating the robustness of our results. Second, our restricted cubic spline dose-response analyses were limited to the range of exposure values derived from individual studies, and the trends observed may be driven by single studies with more extreme exposure values. Thus, a larger number of homogeneous studies across a wide range of exposure values are needed to confirm these results in the future. Third, as an inherent issue in meta-analyses, our analyses combined risk estimates across studies with different designs, populations, settings, statistical adjustments of covariates, etc. (some of which were explored in our subgroup analyses), which likely contributed to heterogeneity in our results. Finally, genetic association studies were not considered. For example, mutations in the *HFE* gene underlying hereditary hemochromatosis (iron overload) have been implicated in several cancers, including breast cancer [99]. Although it was beyond the scope of this review, inclusion of such studies, as well as a closer examination of possible iron-gene interactions, may provide a more complete picture of iron’s role in breast cancer etiology.

## Conclusions

This systematic review and meta-analysis suggests that heme iron intake and serum iron levels may be positively associated with breast cancer risk, whereas no associations were found for intakes of dietary, supplemental, or total iron, or serum/plasma levels of ferritin, TIBC, or TSAT. Although the increases in risk were modest, our findings may have public health implications given the widespread consumption of (heme) iron-rich foods. In light of the methodological and research gaps identified in our review (e.g., consideration of different sources/subtypes of dietary iron intake, comprehensive assessment of supplemental and total iron intake, repeated measures of iron intake/status at multiple time points, exploration of nonlinear trends, stratification of results by menopausal and hormone receptor status), further research is needed to better elucidate the association between iron intake/status and risk of breast cancer.

## Additional files

Additional file 1: Electronic database search strategy. (DOCX 17 kb)

Additional file 2: NOS coding manuals for study quality assessment. (DOCX 21 kb)

Additional file 3: **Table S1.** Quality of included studies assessed using the NOS. (DOCX 29 kb)

Additional file 4: **Figures S1 and S2.** Linear dose-response analyses of associations between iron intake/status and breast cancer risk. (PDF 298 kb)

Additional file 5: **Figures S3 and S4.** Funnel plots for the evaluation of publication bias. (PDF 307 kb)

Additional file 6: **Figures S5–S8.** Sensitivity analyses investigating the influence of individual studies. (PDF 210 kb)

## Abbreviations

BBD: Benign breast disease
BMI: Body mass index
CI: Confidence interval
CRP: C-reactive protein
DL: DerSimonian and Laird
ER: Estrogen receptor
FFQ: Food frequency questionnaire
HRT: Hormone replacement therapy
MOOSE: Meta-analysis of observational studies in epidemiology
NOS: Newcastle-Ottawa Scale
OC: Oral contraceptive
PR: Progesterone receptor
PRISMA: Preferred reporting items for systematic reviews and meta-analyses
ROS: Reactive oxygen species
RR: Relative risk
SD: Standard deviation
TfR: Transferrin receptor
TIBC: Total iron-binding capacity
TSAT: Transferrin saturation
