# Supplementary material for: Iron intake, body iron status, and risk of breast cancer: a systematic review and meta-analysis
Source: BMC Cancer. 2019 Jun 6;19:543. doi: 10.1186/s12885-019-5642-0 (PMC6555759; doi:10.1186/s12885-019-5642-0)
Supplement: Supplementary file 1 — Electronic database search strategy. (DOCX 17 kb) [file 12885_2019_5642_MOESM1_ESM.docx]

**Electronic Database Search Strategy**

**MEDLINE** (Ovid)

Ovid MEDLINE Epub Ahead of Print, In-Process & Other Non-Indexed Citations, Ovid MEDLINE(R) Daily and Ovid MEDLINE(R) <1946 to December 31, 2018>

1. Iron/
2. Iron Compounds/
3. Ferric Compounds/
4. Ferrous Compounds/
5. Iron, Dietary/
6. (iron or fe or ferrum or ferric or ferrous).ti,ab,rn,nm,kf.
7. exp Ferritins/
8. (ferritin* or isoferritin* or apoferritin*).ti,ab,rn,nm,kf.
9. Transferrins/ or Transferrin/ or Receptors, Transferrin/
10. (transferrin or transferrins or TfR* or sTfR*).ti,ab,rn,nm,kf.
11. (TIBC or UIBC).ti,ab,kf.
12. or/1-11
13. exp Breast/ or Breast Diseases/
14. Neoplasms/ or Carcinoma/ or Adenocarcinoma/ or "Neoplasms, Ductal, Lobular, and Medullary"/ or Carcinoma, Ductal/ or Carcinoma, Medullary/
15. 13 and 14
16. exp Breast Neoplasms/
17. exp Carcinoma, Intraductal, Noninfiltrating/
18. Carcinoma, Lobular/
19. ((breast* or mamma or mammary or nipple*) adj15 (cancer* or neoplas* or tumo?r* or carcino* or adenocarcino* or malign* or metasta*)).ti,ab,kf.
20. or/15-19
21. 12 and 20
22. animals/ not humans.sh.
23. 21 not 22
24. remove duplicates from 23

**EMBASE** (Ovid)

Embase Classic + Embase <1947 to December 31, 2018>

1. iron/
2. iron derivative/
3. ferric ion/
4. ferrous ion/
5. iron intake/
6. iron blood level/
7. (iron or fe or ferrum or ferric or ferrous).ti,ab,rn,kw.
8. ferritin/ or ferritin blood level/
9. (ferritin* or isoferritin* or apoferritin*).ti,ab,rn,kw.
10. transferrin/ or transferrin blood level/ or transferrin receptor/
11. (transferrin or transferrins or TfR* or sTfR*).ti,ab,nm,kw.
12. iron binding capacity/
13. (TIBC or UIBC).ti,ab,kw.
14. or/1-13
15. exp breast/ or breast disease/
16. neoplasm/ or carcinoma/ or adenocarcinoma/ or medullary carcinoma/
17. 15 and 16
18. exp breast tumor/
19. ((breast* or mamma or mammary or nipple*) adj15 (cancer* or neoplas* or tumo?r* or carcino* or adenocarcino* or malign* or metasta*)).ti,ab,kw.
20. or/17-19
21. 14 and 20
22. (exp animal/ or nonhuman/) not exp human/
23. 21 not 22
24. remove duplicates from 23

**CINAHL** (EBSCOhost)

Cumulative Index to Nursing and Allied Health Literature (CINAHL) and CINAHL Plus with Full Text <1937 to December 31, 2018>

1. (MH "Iron")
2. (MH "Iron Compounds")
3. (MH "Ferric Compounds")
4. (MH "Ferrous Compounds")
5. (TI (iron or fe or ferrum or ferric or ferrous)) OR (AB (iron or fe or ferrum or ferric or ferrous)) OR (SU (iron or fe or ferrum or ferric or ferrous))
6. (MH "Ferritin")
7. (TI (ferritin* or isoferritin* or apoferritin*)) OR (AB (ferritin* or isoferritin* or apoferritin*)) OR (SU (ferritin* or isoferritin* or apoferritin*))
8. (MH "Transferrin")
9. (TI (transferrin or transferrins or TfR* or sTfR*)) OR (AB (transferrin or transferrins or TfR* or sTfR*)) OR (SU (transferrin or transferrins or TfR* or sTfR*))
10. (TI (TIBC or UIBC)) OR (AB (TIBC or UIBC)) OR (SU (TIBC or UIBC))
11. S1 OR S2 OR S3 OR S4 OR S5 OR S6 OR S7 OR S8 OR S9 OR S10
12. (MH "Breast+") OR (MH "Breast Diseases")
13. (MH "Neoplasms") OR (MH "Adenocarcinoma") OR (MH "Carcinoma") OR (MH "Neoplasms, Ductal, Lobular, and Medullary") OR (MH "Carcinoma, Ductal")
14. S12 AND S13
15. (MH "Breast Neoplasms+") OR (MH "Carcinoma, Lobular")
16. (TI ((breast* or mamma or mammary or nipple*) n15 (cancer* or neoplas* or tumo#r* or carcino* or adenocarcino* or malign* or metasta*))) OR (AB ((breast* or mamma or mammary or nipple*) n15 (cancer* or neoplas* or tumo#r* or carcino* or adenocarcino* or malign* or metasta*))) OR (SU ((breast* or mamma or mammary or nipple*) n15 (cancer* or neoplas* or tumo#r* or carcino* or adenocarcino* or malign* or metasta*)))
17. S14 OR S15 OR S16
18. S11 AND S17
19. ((MH "Rodents+") OR (MH "Animals+")) NOT (MH "Human")
20. S18 NOT S19

**Scopus** (Elsevier)

Scopus <1950 to December 31, 2018>

((TITLE-ABS-KEY (iron OR fe OR ferrum OR ferric OR ferrous OR ferritin* OR isoferritin* OR apoferritin* OR transferrin OR transferrins OR TfR* OR sTfR* OR TIBC OR UIBC)) AND TITLE-ABS-KEY ((breast* OR mamma OR mammary OR nipple*) W/15 (cancer* OR neoplas* OR tumo?r* OR carcino* OR adenocarcino* OR malign* OR metasta*))) AND NOT (KEY (animal* OR nonhuman* OR non-human* OR rodent* OR rat OR rats OR mouse OR mice OR murine OR mus))
