# Supplementary material for: Iron intake, body iron status, and risk of breast cancer: a systematic review and meta-analysis
Source: BMC Cancer. 2019 Jun 6;19:543. doi: 10.1186/s12885-019-5642-0 (PMC6555759; doi:10.1186/s12885-019-5642-0)
Supplement: Supplementary file 2 — NOS coding manuals for study quality assessment. (DOCX 21 kb) [file 12885_2019_5642_MOESM2_ESM.docx]

**Newcastle-Ottawa Scale (NOS) Coding Manuals for Study Quality Assessment**

Adapted from: Wells GA, Shea B, O’Connell D, et al. The Newcastle-Ottawa Scale (NOS) for assessing the quality of nonrandomised studies in meta-analyses. (http://www.ohri.ca/programs/clinical_epidemiology/oxford.asp)

**COHORT STUDIES**

Note: A study can be awarded a maximum of one star (☆) for each numbered item within the Selection and Outcome categories. A maximum of two stars can be given for Comparability. (Max. total score: 9)

***Selection***

1. **Representativeness of the exposed cohort**
2. Truly representative of the average female population in the community ☆
3. Somewhat representative of the average female population in the community ☆
4. Selected group of individuals (e.g., nurses, volunteers, those with specific dietary restrictions or chronic conditions)
5. No description of the derivation of the cohort
6. **Selection of the non-exposed cohort**

*Note: in the case of a continuous exposure, consider individuals with low vs. high levels*

1. Drawn from the same community as the exposed cohort ☆
2. Drawn from a different source
3. No description of the derivation of the non-exposed cohort
4. **Ascertainment of exposure**
5. Secure record (e.g., medical records, biological measurements) ☆
6. Structured interview (e.g., 24-hour dietary recalls, interviewer-administered questionnaire) ☆
7. Written self-report (e.g., self-administered food frequency questionnaire)
8. No description
9. **Demonstration that outcome of interest was not present at start of study**
10. Yes ☆
11. No

***Comparability***

1. **Comparability of cohorts on the basis of the design or analysis (max: 2 stars)**

*Note: Either exposed and non-exposed individuals must be matched in the design and/or confounders must be adjusted for in the analysis. Statements of no differences between groups or that differences were not statistically significant are not sufficient for establishing comparability.*

1. Study controls for age ***and*** total energy/caloric intake (when the exposure is total, dietary, heme, or non-heme iron intake); study controls for age (when the exposure is supplemental iron intake or any measure of body iron status) ☆
2. Study controls for ***at least three*** of the following: body mass index, physical activity, alcohol consumption, family history of breast cancer, history of benign breast disease, oral contraceptive use, hormone replacement therapy use, age at menarche, parity, age at first pregnancy or first live birth, menopausal status, age at menopause ☆
3. No relevant adjustments for confounding

***Outcome***

1. **Assessment of outcome**
2. Independent blind assessment or confirmation of the outcome by reference to secure records (e.g., medical records, pathology reports, histological confirmation) ☆
3. Record linkage (e.g., cancer registry) ☆
4. Self-report with no reference to original medical records to confirm the outcome
5. No description
6. **Was follow-up long enough for outcome to occur**
7. Yes (≥10 years) ☆
8. No (<10 years)
9. **Adequacy of follow-up of cohorts**
10. Complete follow-up (all subjects accounted for) ☆
11. Subjects lost to follow-up unlikely to introduce bias – small number lost (≥90% follow-up), or description provided of those lost ☆
12. Follow-up rate <90% and no description of those lost
13. No statement

**CASE-CONTROL STUDIES**

Note: A study can be awarded a maximum of one star (☆) for each numbered item within the Selection and Exposure categories. A maximum of two stars can be given for Comparability. (Max. total score: 9)

***Selection***

1. **Is the case definition adequate?**
2. Yes, with independent validation (e.g., reference to primary record source such as medical/hospital records, pathology reports, or histological confirmation) ☆
3. Record linkage or self-report with no reference to primary record
4. No description
5. **Representativeness of cases**
6. Consecutive or obviously representative series of cases (i.e., all eligible cases with outcome of interest over a defined period of time, all cases in a defined catchment area, all cases in a defined hospital or clinic, group of hospitals, cancer registry, existing cohort, or an appropriate sample of those cases [e.g., random sample]) ☆
7. Potential for selection bias (not satisfying requirements in part a)
8. No description
9. **Selection of controls**
10. Community controls (i.e., same community as cases and would be cases if had outcome) or controls selected within the same cohort for a nested case-control or case-cohort study ☆
11. Hospital controls or other selected groups of controls (e.g., family members, hospital staff)
12. No description
13. **Definition of controls**
14. No history of outcome (breast cancer) ☆
15. No mention of history of outcome

***Comparability***

1. **Comparability of cases and controls on the basis of the design or analysis (max: 2 stars)**

*Note: Either cases and controls must be matched in the design and/or confounders must be adjusted for in the analysis. Statements of no differences between groups or that differences were not statistically significant are not sufficient for establishing comparability.*

1. Study controls for age ***and*** total energy/caloric intake (when the exposure is total, dietary, heme, or non-heme iron intake); study controls for age (when the exposure is supplemental iron intake or any measure of body iron status) ☆
2. Study controls for ***at least three*** of the following: body mass index, physical activity, alcohol consumption, family history of breast cancer, history of benign breast disease, oral contraceptive use, hormone replacement therapy use, age at menarche, parity, age at first pregnancy or first live birth, menopausal status, age at menopause ☆
3. No relevant adjustments for confounding

***Exposure***

1. **Ascertainment of exposure**
2. Secure record (e.g., medical records, biological measurements) ☆
3. Structured interview where blind to case/control status ☆
4. Interview not blinded to case/control status
5. Written self-report only (e.g., self-administered food frequency questionnaire)
6. No description
7. **Same method of ascertainment for cases and controls**
8. Yes ☆
9. No
10. **Non-response rate**
11. Same or similar rate for both groups ☆
12. Rate different and non-respondents described – unlikely to introduce bias ☆
13. Rate different and no description of non-respondents
14. Response rates not stated
