# Supplementary material for: Iron intake, body iron status, and risk of breast cancer: a systematic review and meta-analysis
Source: BMC Cancer. 2019 Jun 6;19:543. doi: 10.1186/s12885-019-5642-0 (PMC6555759; doi:10.1186/s12885-019-5642-0)
Supplement: Supplementary file 3 — Table S1. Quality of included studies assessed using the NOS. (DOCX 29 kb) [file 12885_2019_5642_MOESM3_ESM.docx]

**Table S1** Quality of included studies assessed using the Newcastle-Ottawa Scale

| **Cohort studies** | **Selection** | | | |  | **Comparability** | |  | **Outcome** | | | **Total score (max: 9)** |
| --- | --- | --- | --- | --- | --- | --- | --- | --- | --- | --- | --- | --- |
|  | **1.**  **Representative-ness of exposed cohort** | **2.**  **Selection of non-exposed cohort** | **3.**  **Ascertainment of exposure** | **4.**  **Absence of outcome at baseline** |  | **5a.**  **Adjusts for age (and energy intake)^a^** | **5b.**  **Adjusts for ≥3 additional confounders^b^** |  | **6.**  **Assessment of outcome** | **7.**  **Length of follow-up**  **(≥10 y)** | **8.**  **Adequacy of follow-up**  **(≥90%)** |  |
| **Iron intake** |  |  |  |  |  |  |  |  |  |  |  |  |
| Kabat et al. 2007 | ☆ | ☆ | - | ☆ |  | ☆ | ☆ |  | ☆ | ☆ | ☆ | 8 |
| Ferrucci et al. 2009 | ☆ | ☆ | - | ☆ |  | ☆ | ☆ |  | ☆ | - | ☆ | 7 |
| Kabat et al. 2010 | ☆ | ☆ | - | ☆ |  | ☆ | ☆ |  | ☆ | - | ☆ | 7 |
| Farvid et al. 2014 | - | ☆ | - | ☆ |  | ☆ | ☆ |  | ☆ | ☆ | ☆ | 7 |
| Farvid et al. 2015 | - | ☆ | - | ☆ |  | ☆ | ☆ |  | ☆ | ☆ | ☆ | 7 |
| Diallo et al. 2016 | - | ☆ | ☆ | ☆ |  | ☆ | ☆ |  | ☆ | ☆ | ☆ | 8 |
| Inoue-Choi et al. 2016 | ☆ | ☆ | - | ☆ |  | ☆ | ☆ |  | ☆ | - | ☆ | 7 |
| **Body iron status** |  |  |  |  |  |  |  |  |  |  |  |  |
| Knekt et al. 1994 | ☆ | ☆ | ☆ | ☆ |  | ☆ | - |  | ☆ | ☆ | - | 7 |
| Herrinton et al. 1995 | ☆ | ☆ | ☆ | ☆ |  | ☆ | - |  | ☆ | ☆ | - | 7 |
| Gaur et al. 2013 | ☆ | ☆ | ☆ | ☆ |  | ☆ | - |  | ☆ | ☆ | ☆ | 8 |
| Wen et al. 2014 | ☆ | ☆ | ☆ | ☆ |  | ☆ | ☆ |  | ☆ | - | - | 7 |
| Chua et al. 2016 | ☆ | ☆ | ☆ | ☆ |  | ☆ | ☆ |  | ☆ | ☆ | ☆ | 9 |
| **Case-control studies^c^** | **Selection** | | | |  | **Comparability** | |  | **Exposure** | | | **Total score (max: 9)** |
|  | **1.**  **Adequacy of case definition** | **2.**  **Representative-ness of cases** | **3.**  **Selection of controls** | **4.**  **Definition of controls** |  | **5a.**  **Adjusts for age (and energy intake)^a^** | **5b.**  **Adjusts for ≥3 additional confounders^b^** |  | **6.**  **Ascertainment of exposure** | **7.**  **Same method of ascertainment** | **8.**  **Non-response rate** |  |
| **Iron intake** |  |  |  |  |  |  |  |  |  |  |  |  |
| Ewertz and Gill 1990 | ☆ | ☆ | ☆ | ☆ |  | ☆ | - |  | - | ☆ | ☆ | 7 |
| Negri et al. 1996 | ☆ | ☆ | - | ☆ |  | ☆ | - |  | - | ☆ | ☆ | 6 |
| Cade et al. 1998 | - | - | - | ☆ |  | ☆ | ☆ |  | - | ☆ | ☆ | 5 |
| Levi et al. 2001 | ☆ | ☆ | - | - |  | ☆ | ☆ |  | - | ☆ | ☆ | 6 |
| Adzersen et al. 2003 | ☆ | ☆ | - | - |  | ☆ | ☆ |  | - | ☆ | - | 5 |
| Michels et al. 2006 | ☆ | - | ☆ | ☆ |  | ☆ | ☆ |  | - | ☆ | - | 6 |
| Hong et al. 2007 | ☆ | ☆ | ☆ | ☆ |  | - | ☆ |  | - | ☆ | ☆ | 7 |
| Kallianpur et al. 2008 | ☆ | ☆ | ☆ | ☆ |  | ☆ | ☆ |  | - | ☆ | ☆ | 8 |
| Moore et al. 2009 | ☆ | - | ☆ | - |  | ☆ | - |  | - | ☆ | - | 4 |
| Bradshaw et al. 2013 | ☆ | ☆ | ☆ | ☆ |  | ☆ | - |  | - | ☆ | - | 6 |
| **Body iron status** |  |  |  |  |  |  |  |  |  |  |  |  |
| Garland et al. 1996 | ☆ | - | ☆ | ☆ |  | ☆ | ☆ |  | ☆ | ☆ | ☆ | 8 |
| Cui et al. 2007 | ☆ | ☆ | ☆ | ☆ |  | ☆ | ☆ |  | ☆ | ☆ | ☆ | 9 |
| Moore et al. 2009 | ☆ | - | ☆ | - |  | ☆ | - |  | ☆ | ☆ | - | 5 |
| Stevens et al. 2011 | - | ☆ | ☆ | ☆ |  | ☆ | - |  | ☆ | ☆ | ☆ | 7 |
| Graff et al. 2014 | ☆ | ☆ | ☆ | ☆ |  | ☆ | ☆ |  | ☆ | ☆ | ☆ | 9 |
| Quintana Pacheco et al. 2018 | ☆ | ☆ | ☆ | ☆ |  | ☆ | ☆ |  | ☆ | ☆ | ☆ | 9 |

^a^ To receive a star, studies assessing dietary, total, heme, and/or non-heme iron intake must have adjusted for both age and total energy intake, whereas all other studies only needed to adjust for age

^b^ To receive a star, the study must have adjusted for at least three of the following: body mass index, physical activity, alcohol consumption, family history of breast cancer, history of benign breast disease, oral contraceptive use, hormone replacement therapy use, age at menarche, parity, age at first pregnancy or first live birth, menopausal status, and age at menopause

^c^ Including traditional case-control, nested case-control, and case-cohort studies
