# Supplementary material for: Iron intake, body iron status, and risk of breast cancer: a systematic review and meta-analysis
Source: BMC Cancer. 2019 Jun 6;19:543. doi: 10.1186/s12885-019-5642-0 (PMC6555759; doi:10.1186/s12885-019-5642-0)
Supplement: Supplementary file 4 — Figures S1 and S2. Linear dose-response analyses of associations between iron intake/status and breast cancer risk. (PDF 298 kb) [file 12885_2019_5642_MOESM4_ESM.pdf]

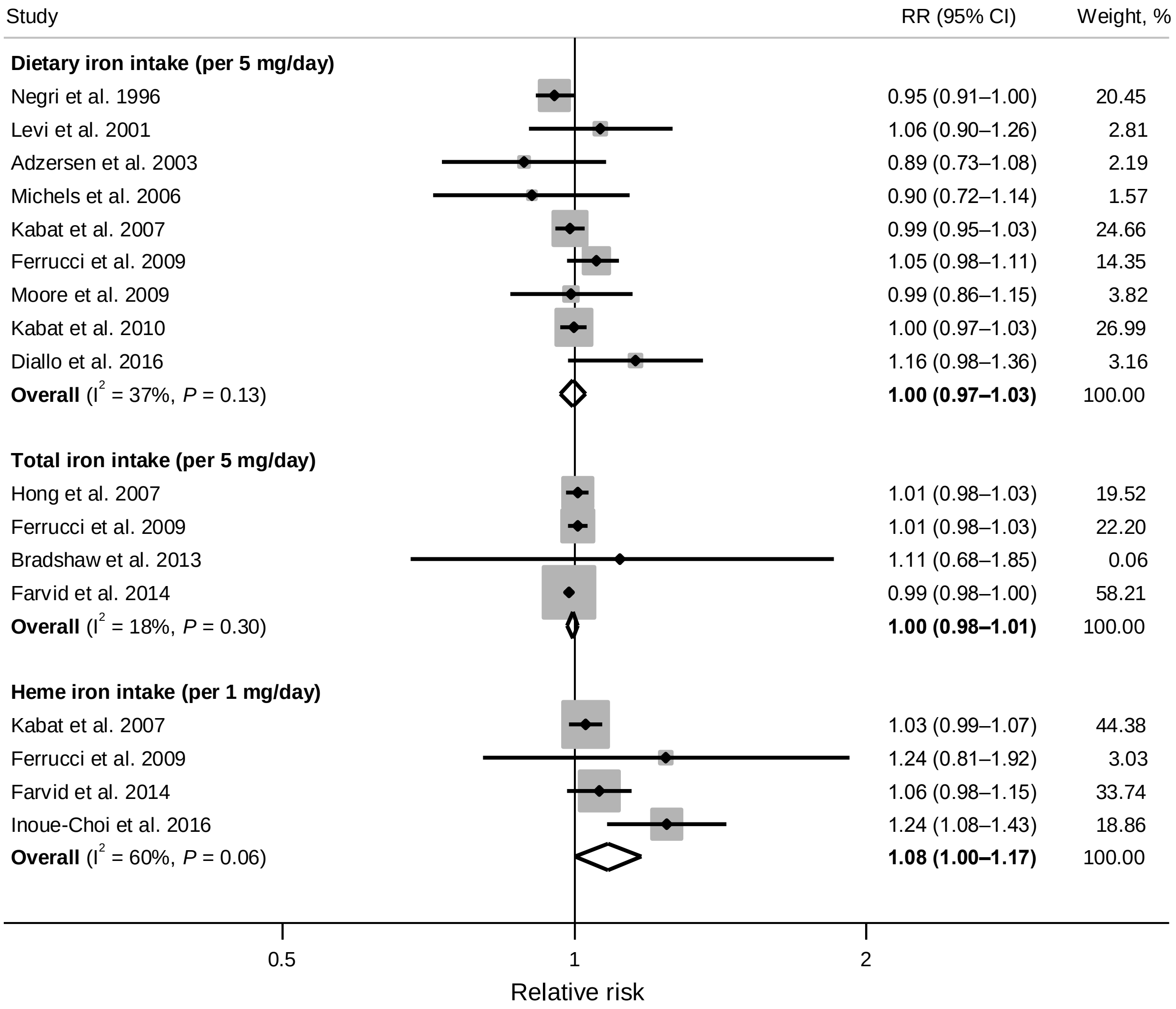

Fig. S1 Forest plot of linear dose-response analyses for the associations between iron intake and breast cancer risk. The diamonds represent the pooled relative risks and corresponding 95% confidence intervals obtained from random-effects meta-analyses. The dots and horizontal lines represent the relative risks and corresponding 95% confidence intervals of individual studies, and the sizes of shaded squares are proportional to the weight contributed by each study to the pooled estimate. I^2 is the proportion of the total variability attributable to between-study heterogeneity, and *P* is from Cochran's Q test evaluating the presence of heterogeneity.

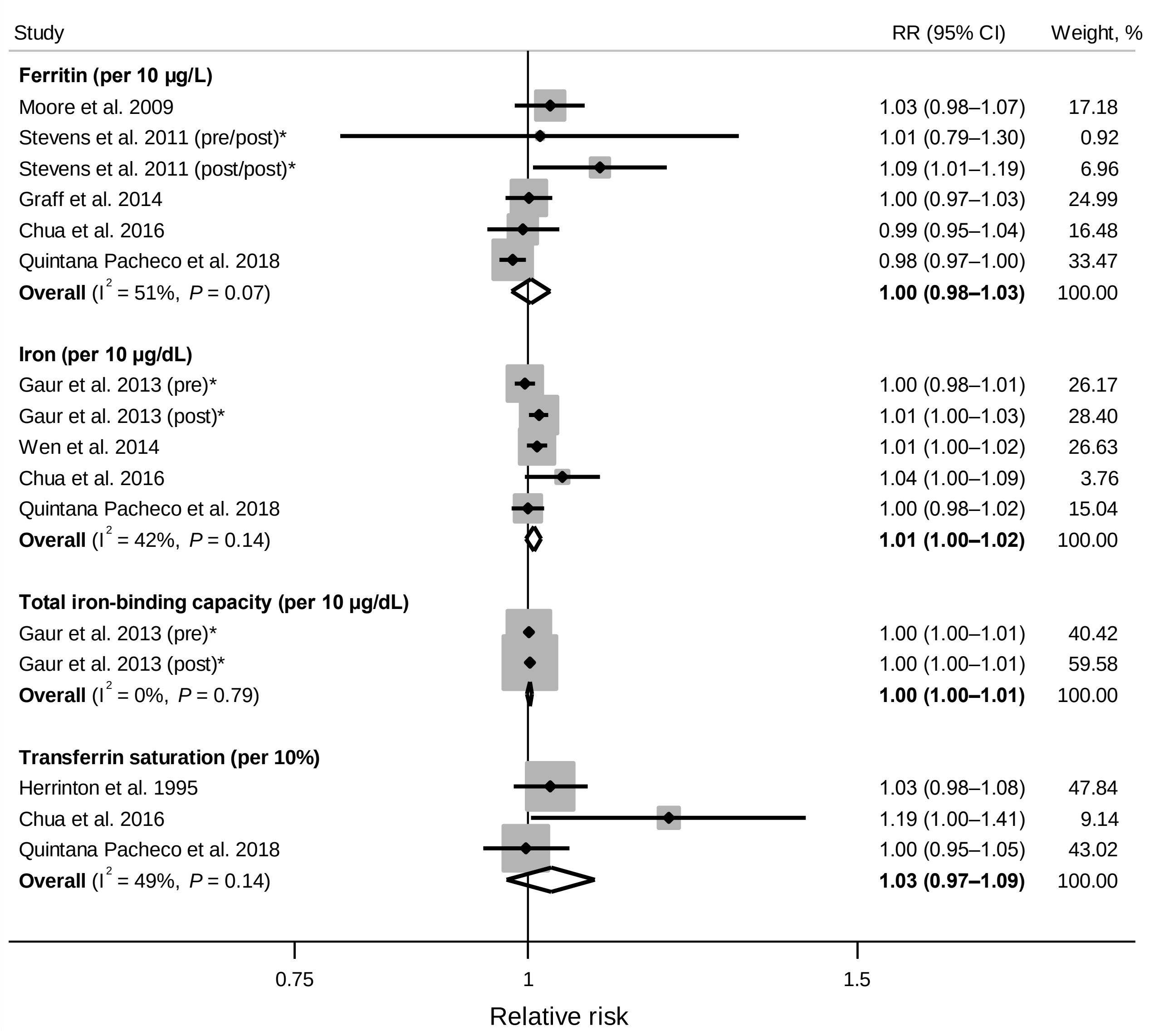

Fig. S2 Forest plot of linear dose-response analyses for the associations between serum/plasma indicators of body iron status and breast cancer risk. The diamonds represent the pooled relative risks and corresponding 95% confidence intervals obtained from random-effects meta-analyses. The dots and horizontal lines represent the relative risks and corresponding 95% confidence intervals of individual studies, and the sizes of shaded squares are proportional to the weight contributed by each study to the pooled estimate. I^2 is the proportion of the total variability attributable to between-study heterogeneity, and *P* is from Cochran's Q test evaluating the presence of heterogeneity. *Stevens et al. 2011 reported separate estimates for premenopausal (pre/post) and postmenopausal (post/post) ferritin levels in relation to postmenopausal breast cancer risk; Gaur et al. 2013 reported separate estimates for premenopausal (pre) and postmenopausal (post) breast cancer.
