# Supplementary material for: Iron intake, body iron status, and risk of breast cancer: a systematic review and meta-analysis
Source: BMC Cancer. 2019 Jun 6;19:543. doi: 10.1186/s12885-019-5642-0 (PMC6555759; doi:10.1186/s12885-019-5642-0)
Supplement: Supplementary file 5 — Figures S3 and S4. Funnel plots for the evaluation of publication bias. (PDF 307 kb) [file 12885_2019_5642_MOESM5_ESM.pdf]

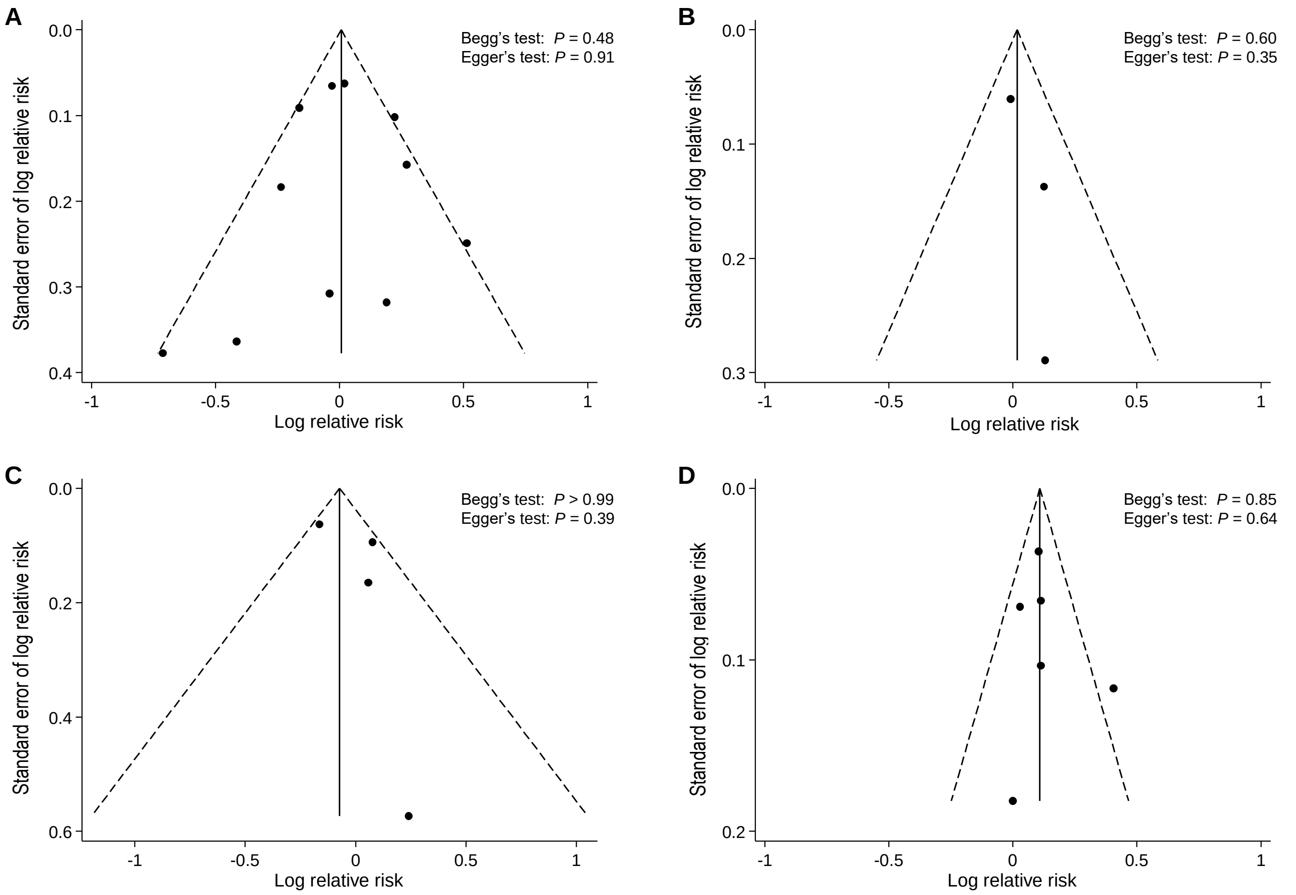


**Fig. S3** Funnel plots for the evaluation of publication bias in the meta-analysis of breast cancer risk in relation to the highest vs. lowest category of **(A)** dietary iron intake; **(B)** supplemental iron intake; **(C)** total iron intake; and **(D)** heme iron intake. The dots represent estimates from individual studies. The vertical solid lines represent pooled effect estimates, and the dashed diagonal lines represent pseudo 95% confidence intervals. All statistical tests are two-sided.

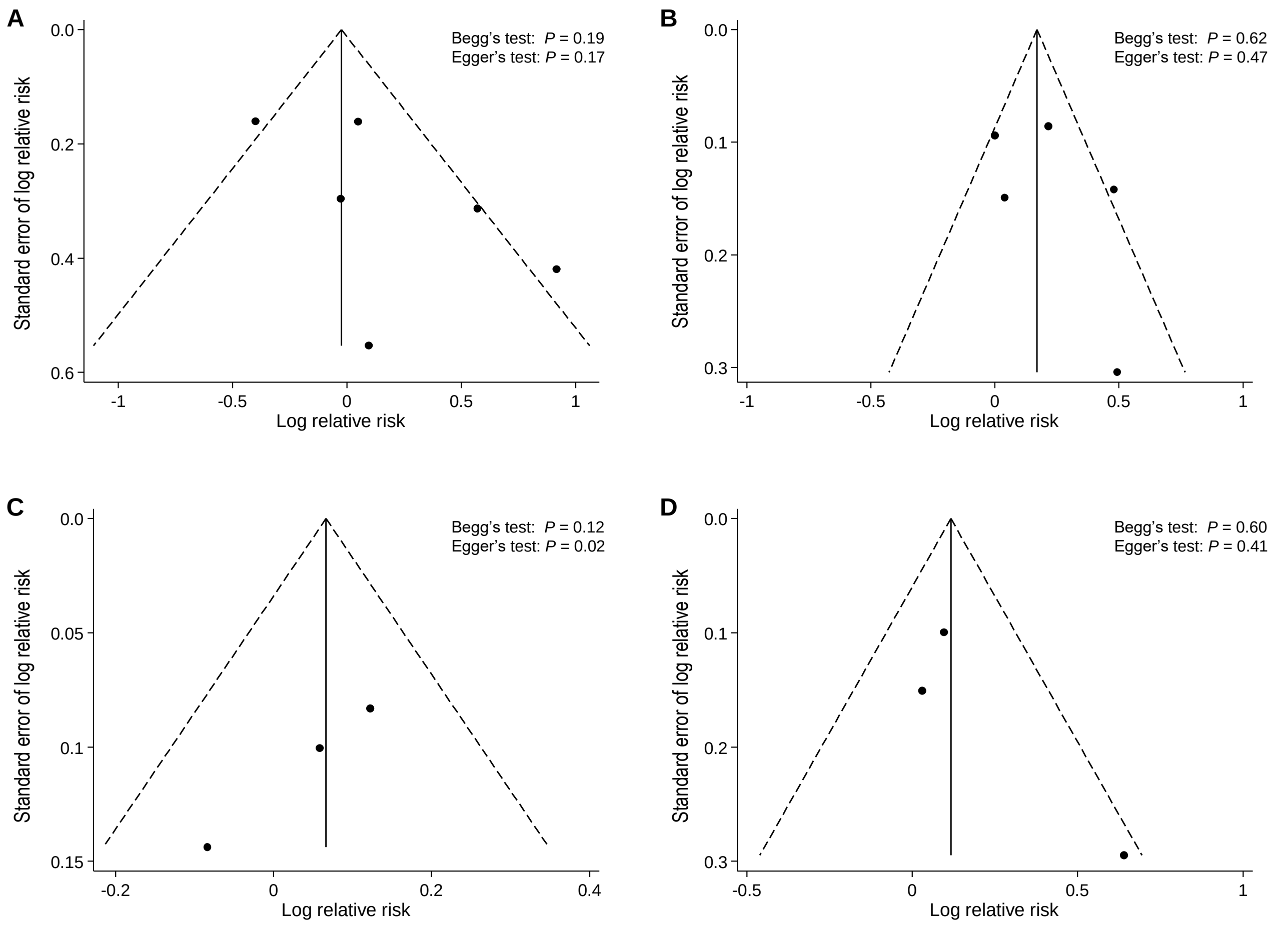


**Fig. S4** Funnel plots for the evaluation of publication bias in the meta-analysis of breast cancer risk in relation to the highest vs. lowest category of serum/plasma **(A)** ferrin; **(B)** iron; **(C)** total iron-binding capacity or transferrin; and **(D)** transferrin saturation. The dots represent estimates from individual studies. The vertical solid lines represent pooled effect estimates, and the dashed diagonal lines represent pseudo 95% confidence intervals. All statistical tests are two-sided.
