# Supplementary material for: Iron intake, body iron status, and risk of breast cancer: a systematic review and meta-analysis
Source: BMC Cancer. 2019 Jun 6;19:543. doi: 10.1186/s12885-019-5642-0 (PMC6555759; doi:10.1186/s12885-019-5642-0)
Supplement: Supplementary file 6 — Figures S5–S8. Sensitivity analyses investigating the influence of individual studies. (PDF 210 kb) [file 12885_2019_5642_MOESM6_ESM.pdf]

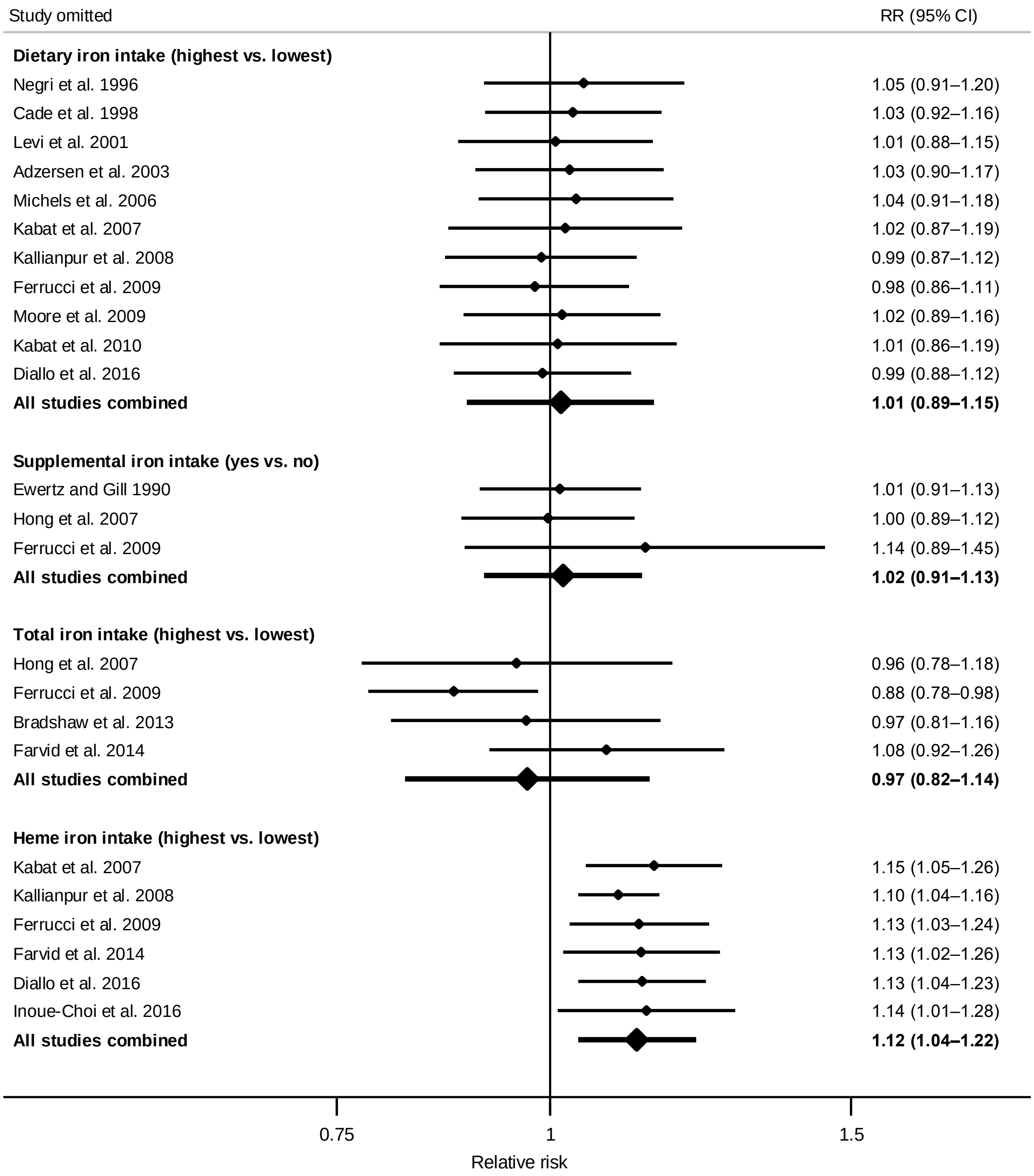

Fig. S5 Sensitivity analysis investigating the influence of individual studies on the pooled effect estimates in the *highest vs. lowest* meta-analyses of dietary, supplemental, total, and heme iron intake in relation to breast cancer risk. The dots and horizontal lines represent the pooled relative risks and corresponding 95% confidence intervals when each study is omitted. The diamonds and thick horizontal lines represent the pooled relative risks and corresponding 95% confidence intervals when all studies are combined (i.e., none omitted).

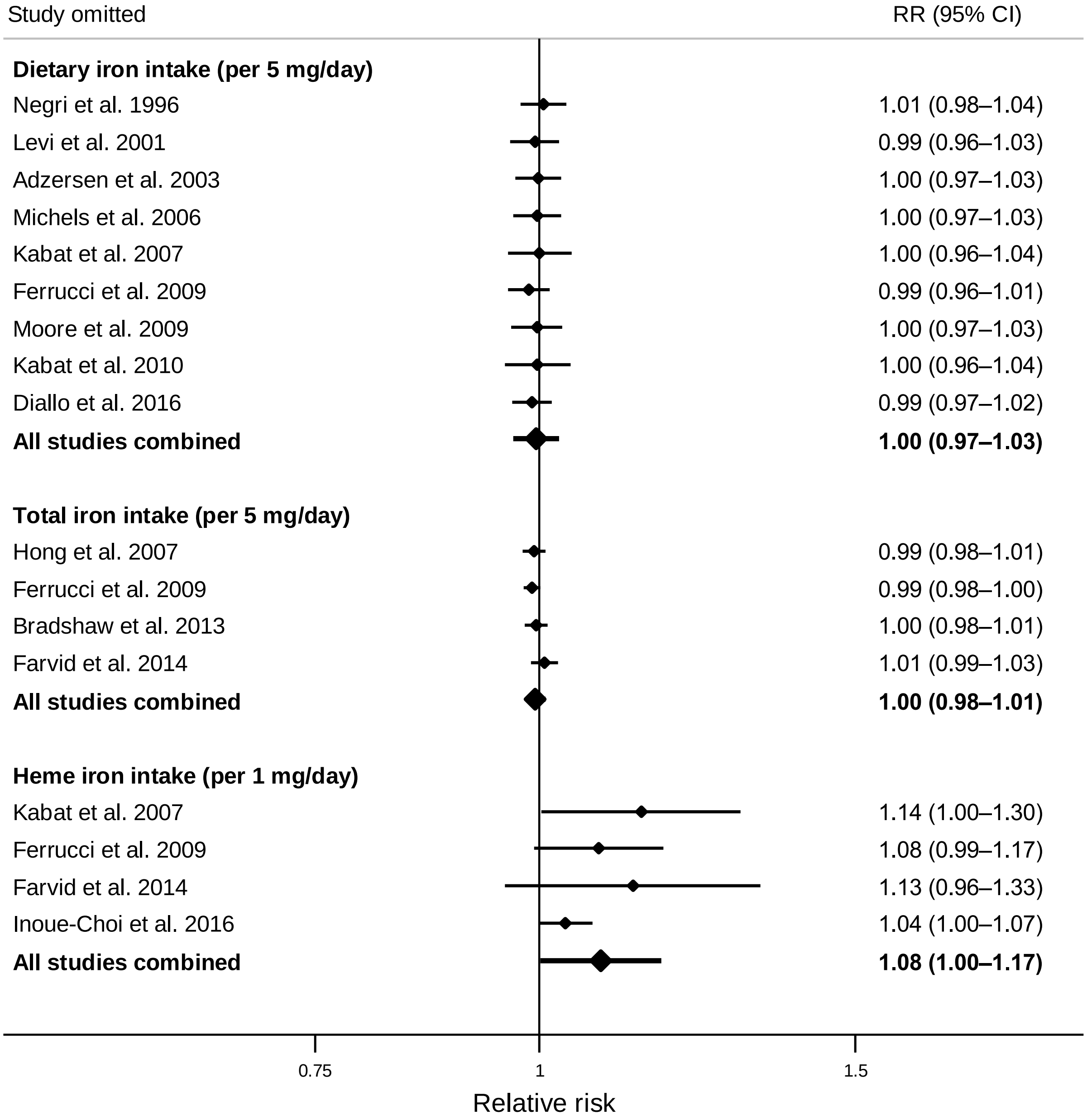

Fig. S6 Sensitivity analysis investigating the influence of individual studies on the pooled effect estimates in the *linear dose-response* meta-analyses of dietary, total, and heme iron intake in relation to breast cancer risk. The dots and horizontal lines represent the pooled relative risks and corresponding 95% confidence intervals when each study is omitted. The diamonds and thick horizontal lines represent the pooled relative risks and corresponding 95% confidence intervals when all studies are combined (i.e., none omitted).

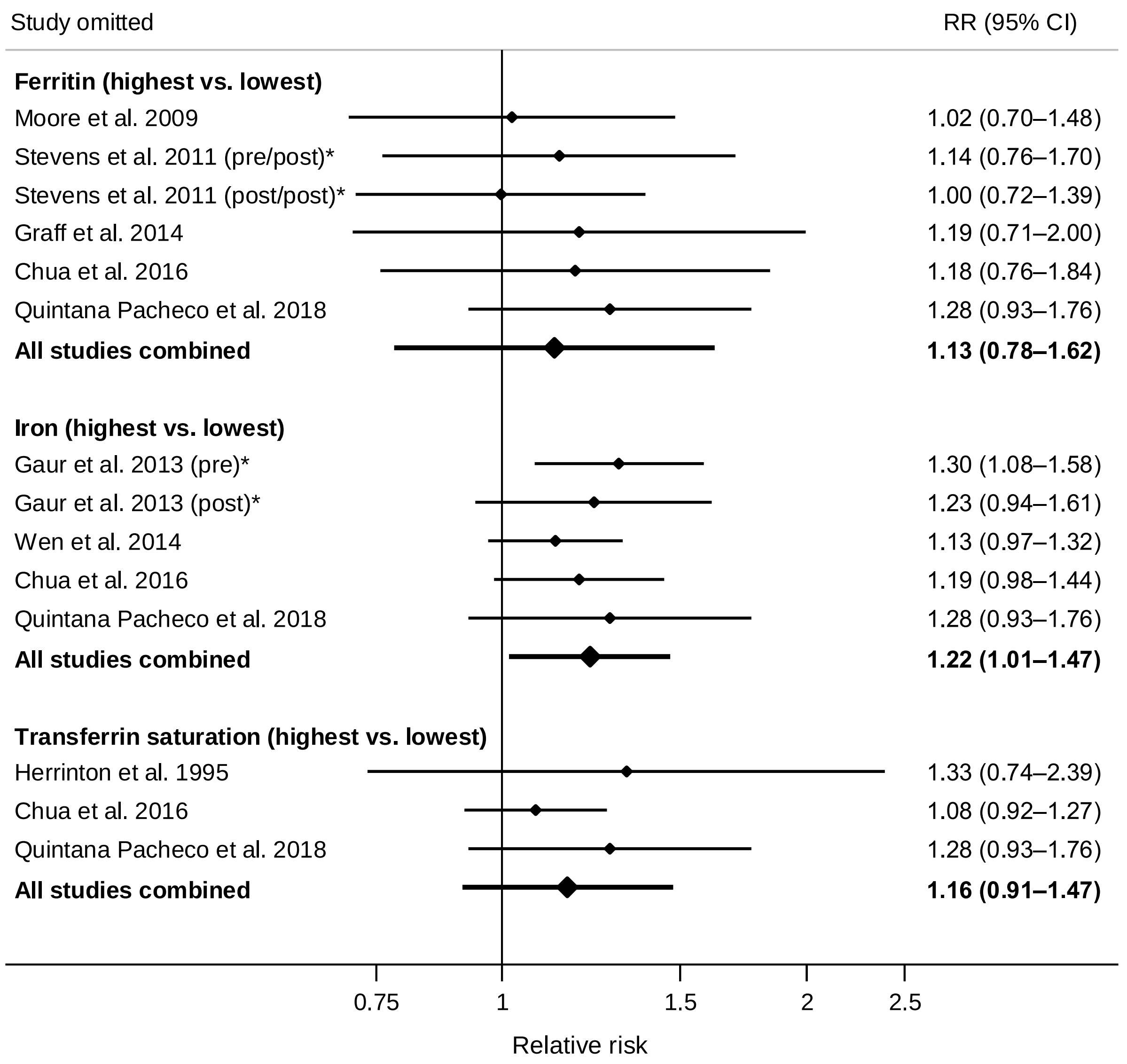

Fig. S7 Sensitivity analysis investigating the influence of individual studies on the pooled effect estimates in the *highest vs. lowest* meta-analyses of serum/plasma ferritin, iron, and transferrin saturation in relation to breast cancer risk. The dots and horizontal lines represent the pooled relative risks and corresponding 95% confidence intervals when each study is omitted. The diamonds and thick horizontal lines represent the pooled relative risks and corresponding 95% confidence intervals when all studies are combined (i.e., none omitted). *Stevens et al. 2011 reported separate estimates for premenopausal (pre/post) and postmenopausal (post/post) ferritin levels in relation to postmenopausal breast cancer risk; Gaur et al. 2013 reported separate estimates for premenopausal (pre) and postmenopausal (post) breast cancer.

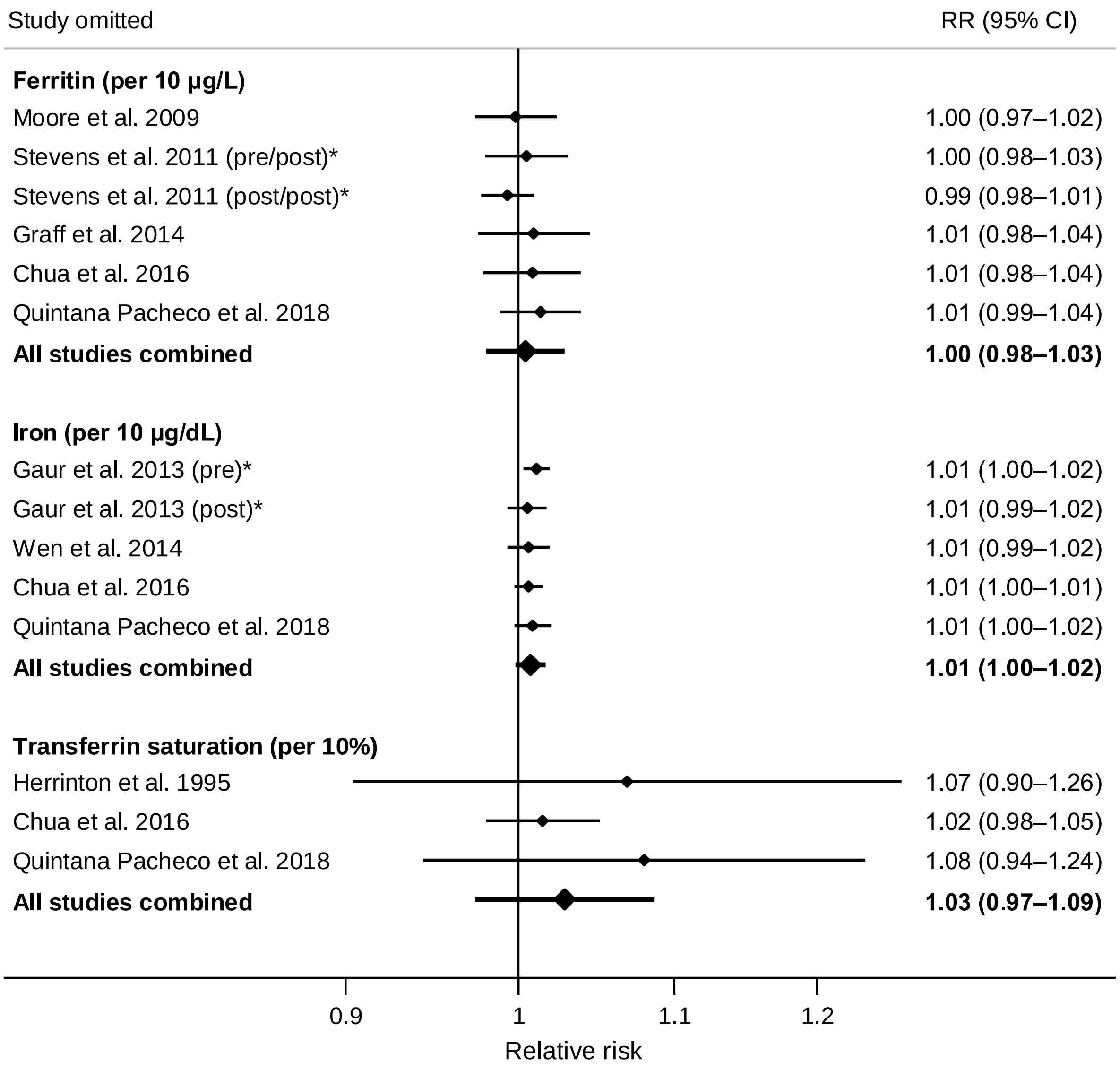

Fig. S8 Sensitivity analysis investigating the influence of individual studies on the pooled effect estimates in the *linear dose-response* meta-analyses of serum/plasma ferritin, iron, and transferrin saturation in relation to breast cancer risk. The dots and horizontal lines represent the pooled relative risks and corresponding 95% confidence intervals when each study is omitted. The diamonds and thick horizontal lines represent the pooled relative risks and corresponding 95% confidence intervals when all studies are combined (i.e., none omitted). *Stevens et al. 2011 reported separate estimates for premenopausal (pre/post) and postmenopausal (post/post) ferritin levels in relation to postmenopausal breast cancer risk; Gaur et al. 2013 reported separate estimates for premenopausal (pre) and postmenopausal (post) breast cancer.
